# Level of health literacy in Latvia and Lithuania: a population-based study

**DOI:** 10.1186/s13690-022-00886-3

**Published:** 2022-07-11

**Authors:** Ieva Gatulytė, Valērija Verdiņa, Zane Vārpiņa, Ágnes Lublóy

**Affiliations:** 1 Stockholm School of Economics in Riga, Riga, Latvia; 2 Corvinus University of Budapest, Budapest, Hungary

**Keywords:** Health literacy, Population-based study, European health literacy survey (HLS-EU), Latvia, Lithuania

## Abstract

**Background:**

Measuring and understanding the level of health literacy serves as a starting point for developing various policies in health care. The consequences of weak health literacy competencies are severe; they result in riskier health behaviour, poorer health status, more frequent emergency visits and hospitalizations. This research has three aims: *i*) measure the level of health literacy in the populations of Latvia and Lithuania; *ii*) investigate which demographic and socioeconomic determinants are associated with it; and *iii*) discuss the means of improving its current level.

**Methods:**

We employ a validated survey tool, the 47-item European Health Literacy Questionnaire (HLS-EU-Q). In addition to the 47 questions in the domains of health care, disease prevention, and health promotion, the participants’ demographic and socioeconomic characteristics are assessed. Face-to-face paper-assisted surveys are conducted with randomly selected residents from Latvia and Lithuania. The level of health literacy is measured by the health literacy index. Spearman correlation analyses and multiple regressions models are employed for investigating the association between the health literacy level and its determinants. The survey tool is complemented with in-depth interviews with six healthcare industry experts in order to assess the most promising ways to improve the level of health literacy.

**Results:**

The stratified random sampling with quota elements assured a representative sample in terms of gender, urban/rural distribution and regions. In Latvia, 79% of the population possesses weak health literacy competencies. In Lithuania, 73% of the population can be characterized with inadequate or problematic level of health literacy. The most important determinants of the health literacy level include age, financial situation, social status, and ethnicity. In particular, elderly (aged 76 and over) and the Latvian-speaking population are less health literate, while those having better financial situation and higher social status are more health literate. The three most promising ways to improve the level of health literacy, as suggested by the healthcare industry experts, include health education in schools, provision of structured health-related information in Latvian and Lithuanian, and guidelines for the most common health problems.

**Conclusions:**

The proportion of population with inadequate or problematic level of health literacy is higher in Latvia and Lithuania than in several other European countries. There is an urgent need to develop policies to improve it.

**Supplementary Information:**

The online version contains supplementary material available at 10.1186/s13690-022-00886-3.

## Background

Health literacy refers to people’s ability to obtain, understand, evaluate and apply health related information in an appropriate way and navigate in sophisticated healthcare systems [1]. Over the last couple of years, health literacy has been gradually gaining attention among researchers, policy makers and the whole society [1, 2]. According to a research conducted in eight European countries by the European Health Literacy Survey (HLS-EU) Consortium, around half of the citizens have poor health literacy skills [1, 3]. The World Health Organization described the situation in Europe as “a health literacy crisis” [2].

The consequences of weak health literacy competencies are severe; they result in less healthier choices, riskier behaviour, poorer health status, lower level of self-management and more hospitalization [2]. As a result, significant amount of human and financial resources are drained from the health system. In contrast, at society level, strong health literacy competencies are associated with longer lives, increased productivity, and enhanced transmission of health knowledge and behaviour to future generations [4]. At individual level, sufficient health literacy allows people to understand and implement the recommendations from their doctors properly and contributes to better quality of life [5]. It is therefore crucial to assess and improve the level of health literacy of a population. Measuring and understanding the level of health literacy serves as a starting point for developing various policies in health care [6].

The aim of this study is threefold. The first objective of this study is to measure the level of health literacy in Latvia and Lithuania which has never been done in the literature before. In particular, we employ the validated European Health Literacy Questionnaire (HLS-EU-Q) and run face-to-face surveys with randomly selected residents from Latvia and Lithuania. The second aim of this study is to investigate the determinants being associated with the level of health literacy in these two Baltic countries. Third, we aim at investigating the means of improving the current level of health literacy in Latvia and Lithuania and formulate policy recommendations for improving it. Here, we take a forward-looking approach and conduct in-depth interviews with healthcare industry experts. Improving the level of health literacy in Latvia and Lithuania is especially important given that the life expectancy and the healthy life years at birth are among the lowest, while preventable and amenable mortality rates are the highest in Latvia and Lithuania when compared to other European countries [7].

In the rest of the Background section, first we show the main socio-demographic characteristics of Latvia and Lithuania. Second, we introduce the concept of health literacy. Third, we review studies measuring the level of health literacy in Europe at population level and show the gap in assessing the level of health literacy in Latvia and Lithuania. Fourth, we discuss previous empirical evidence on the factors being associated with the level of health literacy. Fifth, we review studies on the implications of limited health literacy in order to demonstrate how important it is to increase the level of health literacy in a society.

### Main sociodemographic characteristics of Latvia and Lithuania

Latvia and Lithuania are small Northern European countries that share many socio-demographic characteristics. In the last two decades populations of the two countries have shrunk due to several waves of emigration and population ageing. In 2020, the size of the population is 1.9 million in Latvia and 2.8 million in Lithuania (Table 1) [8]. The median age (Latvia 43.7 years, Lithuania 44.2 years) is close to the EU average, but the populations are ageing faster than EU as a whole [8]. Although the living standards improved considerably in both countries in the past decades, their levels have not reached that in the Western European countries. In 2020 the GDP per capita stood at 69% and 84% of the EU average in Latvia and Lithuania, respectively [8].

**Table 1:** Socio-demographic indicators of Latvia and Lithuania in comparison to EU average

	Year	Latvia	Lithuania	EU27 (2020) / EU28 (2019)
Population size, millions	2020	1.9	2.8	447.3
Median age of population	2020	43.7	44.2	43.9
GDP per capita, percent of EU average	2020	69%	84%	100%
Life expectancy at birth in years, total	2019	75.7	76.5	81.3
Life expectancy at birth females	2019	80.1	81.2	84
Life expectancy at birth males	2019	70.9	71.6	78.5
Healthy Life Years, total	2019	53.1	57.5	64.6
Healthy Life Years females	2019	54.1	59.1	65.1
Healthy Life Years males	2019	52.2	56	64.2
Health care expenditure, EUR per capita	2019	1046	1223	3102

*Source:* Eurostat (2021) database [8]

Life expectancy in both countries is among the lowest in the EU, and no significant improvements are observed in this domain [8]. In Latvia, female life expectancy at birth lags 4 years behind EU average (3 years in Lithuania), but for males the difference is still larger – almost 8 years in Latvia and 7 years in Lithuania [8]. Moreover, healthy life years (HLYE) at birth, indicator that measures the number of remaining years that a person of specific age is expected to live without any severe or moderate health problems, is 53 years in Latvia, the lowest in EU (average 65 years), and more than 20 years below that of Sweden (highest in the EU) [8]. Lithuania has a slightly better situation in terms of HLYE, but the measure is still well below the EU average. In both countries, per capita health care spending is about one third of the EU level (Table 1) [8].

### The concept of health literacy

Since the introduction of the term *health literacy* in the 1970s, the concept gained its relevance in healthcare across the world [9]. Initially, health literacy was concerned only with the capabilities to read as well as perceive health-related information [10]. Gradually, the term encompassed several other dimensions. According to Batterham et al. [10], the definition of health literacy concentrates on various aspects of the individual’s capabilities to “*access, understand and use health information from many sources*” (p. 4). The health literacy classification suggested by Nutbeam [5] divides the concept of health literacy into three dimensions: functional literacy, interactive literacy, and critical literacy. *Functional literacy* focuses mostly on literacy and numerical skills that are essential in everyday life. *Interactive literacy* broadens the definition of health literacy by including not only the literacy skills but also the cognitive skills. Cognitive skills help to understand the different types of information better and support its use in various situations. *Critical literacy* helps to analyse the information in a critical way and stimulates its application to different situations in life [5].

Although in the literature there is no agreed definition of the term *health literacy*, the broad formulation by Sørensen et al. [9] is widely used to capture health literacy: “*Health literacy is linked to literacy and entails people’s knowledge, motivation and competences to access, understand, appraise, and apply health information in order to make judgements and take decisions in everyday life concerning health care, disease prevention and health promotion to maintain or improve quality of life during the life course*” (p. 3). The definition considers all the above-mentioned concepts of health literacy; therefore, this notion is extensively used when analysing questions related to health and healthcare. Moreover, this formulation includes two essential perspectives of health literacy. The first perspective is associated with the cognitive abilities of a person related to medicine, while the second aspect takes into account wider skills linked to health and healthcare [9]. The first perspective can be perceived as *medical literacy* focusing on a person’s abilities and knowledge related to his/her own health [11]. These abilities include numerical and reading skills being essential to operate in the healthcare environment [12]; ability to read and interpret information related to health [13]; comprehension of diagnoses, medicine and health-related instructions as well as the capability to follow them [14]; and participation in decision-making together with health professionals [15]. The second essential perspective of health literacy is related to a person’s ability to access, process, and interpret the health-related information, and make decisions based on it [16]. Note that the broad definition of health literacy, as formulated in [9], also includes public health elements.

### Level of health literacy in Europe

In the literature, relatively few population-based studies estimate the level of health literacy on a comparative scale. In Europe, the most prominent research was conducted by Sørensen and his colleagues on behalf of the HLS-EU Consortium in 2011 [1]. Eight European countries (Austria, Bulgaria, Germany, Greece, Spain, Ireland, the Netherlands, Poland) participated in the European Health Literacy Survey, providing reliable estimates for the health literacy levels across the sample countries. Overall, around 8000 respondents were included in the survey with a minimum age limit of 15 years [1].

According to the study, limited health literacy is a prevailing problem in Europe [1]. Almost half of the participants (approximately 47%) are subject to an insufficient level of health literacy: 12% have an inadequate level of general health literacy, and more than one third (35%) of the respondents can be characterized by a problematic health literacy level. The authors find that 17% of the study population possesses excellent health literacy skills while the remaining 36% have a sufficient level. As a result, they conclude that a little over half of the survey participants have a decent level of health literacy in Europe.

Nevertheless, there are significant differences across the countries studied [1]. The Netherlands stands out with the highest health literacy level in Europe. As high as 25.1% of the Dutch have excellent health literacy, and less than a third (28.7%) have limited health literacy (inadequate and problematic taken together), of which only 1.8% is inadequate. In contrast, the Bulgarian population possesses the lowest health literacy level in Europe – close to third (62.1%) of the Bulgarians have limited health literacy, and more than a quarter can be characterized by inadequate health literacy levels [1].

Paakkari, Torppa, Mazur et al. [17] have recently assessed the level of health literacy in several European countries. The population-based survey was run from 2017 to 2018. The study measured and compared health literacy among 15-year-old adolescents in ten European countries (Austria, Belgium, Czech Republic, UK, Estonia, Finland, Germany, Macedonia, Poland, and Slovakia). A different method – Health Literacy for School-Aged Children instrument – was used to measure the adolescents’ subjective health literacy. The results are hence not directly comparable with the Sørensen et al. [1] study. The authors in [17] distinguish three categories: low, moderate and high health literacy. According to their findings, adolescents in Finland and Macedonia possess high levels of health literacy (close to 40%), while in other countries it ranges between 12.8 and 19.2%. The highest shares of low health literacy were documented in Czechia, Austria and Germany – 17.7%, 16.6% and 16%, respectively. The proportion of adolescents with low-level of health literacy is the lowest in Macedonia (6.0%), Finland (9.0%) and Poland (9.3%).

A few studies concentrate on particular countries and particular subgroups within the population in Europe. For example, Vozikis, Drivas, and Milioris [18] investigated the level of health literacy among Greek students and found that their level of health literacy is decent, and they can be considered as healthy individuals.

Another study investigating the health literacy level was conducted in Germany [19]. The authors surveyed 2000 participants and reported that 47.3% of the respondents aged 15–29 years showed limited perceived health literacy, while 66.4% aged 65 years and older had limited perceived health literacy. The study in Turkey, based on a representative population survey using the HLS-EU-Q questionnaire, found that the health literacy level was inadequate for 25.9% of the population, problematic for 41.4%, adequate for 23.6%, and excellent for 9.1%. of the Turks [20].

There is limited or no information about the level of health literacy in other European countries, including Latvia and Lithuania. These two Baltic countries have not been part of any collaborative research initiative assessing the health literacy skills of the population. In addition, to the authors’ knowledge, no other study measured the level of health literacy in these populations.

### Determinants of health literacy

The level of health literacy is determined by a number of factors that can be divided into two categories: *demographic* and *socioeconomic determinants* [3]. Demographic determinants include, primarily, *gender* and *age.* Literature offers mixed evidence on *gender* as a determinant of health literacy [1, 21–26]. A number of studies find a statistically significant relationship between gender and health literacy, and women tend to have somewhat a higher health literacy level [1, 22–24]. Empirically higher health literacy for women has been shown, for example, in the European population [1]; in the senior population [22], among Korean adults [23]; and among Dutch adults [24]. In contrast, other studies conclude that the level of health literacy is not associated with gender [25, 26]. Older *age* of the individual is shown to be negatively linked with health literacy in a number of studies [2, 19, 27]. For Europe, in the European Health Literacy Survey, it was found that the level of health literacy is negatively associated with age in Greece, Bulgaria, Poland and Spain, while this association was insignificant in Germany and Ireland [1]. Baker et al. [28] provide several explanations for the negative association. In particular, older people tend to have a lower health literacy level because of deteriorating cognitive functions during the lifetime. Their ability to extract, analyse, and memorize information weakens, which leads to decreased capacity to deal with health-related information [28].

Previous studies showed that socioeconomic indicators are stronger predictors of health literacy level compared to demographic indicators. *Education*, *income, social status*, and *employment* are essential determinants of health literacy [2, 25–27, 29]. Specifically, more *educated* people tend to have a higher level of health literacy [2, 25–27]. Based on a systematic review of 85 scientific articles in the US, Paasche-Orlow et al. [25] confirm unambiguously that a higher level of education is associated with a higher level of health literacy. They found that people who graduated from high schools in the top quartile were less subject to a low health literacy level. In the European Health Literacy Survey, the strongest correlations between the level of health literacy and education were documented in Greece, Bulgaria, Poland and Spain, which illustrates that countries with lower levels of education also have lower levels of health literacy [3]. However, in countries where the level of education is high, the correlations were weaker – these countries are Germany, the Netherlands, and Austria [3]. Since the level of health literacy is based on people’s competence in accessing, understanding, appraising, and applying health-related information, education plays a major role [2]. More educated people tend to make more informed health decisions since they can process and assess health-related information more accurately. However, as noted by the WHO, even in countries with advanced economies and high-quality education, the problem of limited health literacy is present [2].

Similar to education, higher *income* is also a determinant of the health literacy level [1, 25, 27]. The meta study of Paasche-Orlow et al. [25] confirms this finding based on US literature. People with higher income do not only have better access to health services but can also be characterized by a healthier lifestyle regarding nutrition, physical activity, tobacco use, and excessive alcohol consumption [30, 31]. At the same time, previous literature finds that individuals with *lower social status* tend to have poorer health literacy than the overall population [1, 2, 19]. One prominent example for individuals with lower social status and, thus, weaker health literacy competencies are migrants. As a general rule, migrants do not have the possibility to get sufficient knowledge about health because of economic, social and language hurdles [2]. The study of Berens et al. [19] found empirical evidence that migrants in Germany tend to have a lower health literacy level as a result of limited language skills which makes it difficult to process available information. In addition, *employment* status is considered to be another determinant of the health literacy level [29]. As it was ascertained by the researchers from HLS-EU Consortium [3], full-time or part-time employees tend to have a higher health literacy level.

Empirical evidence shows that the level of health literacy is influenced by the *frequency of health service usage* [1, 19, 32, 33]. According to Sørensen et al. [1], limited health literacy is more common among people who perceive their health status as bad or very bad, have more than six doctor appointments per year, are older, and have lower socioeconomic status. Berens et al. [19] also showed that limited health literacy (both perceived and functional) is associated with higher frequency of doctor visits. Two other studies found a causal link between health literacy competencies and the frequency of health services usage. Netemeyer et al. [32] documented statistically significant direct effects from health literacy on the frequency of physician consultations, controlling for physical health, age, and socioeconomic factors. Vandenbousch et al. [33] report that low health literacy increases the use of health services, including more frequent and longer hospitalizations, more general practitioner home consultations, and emergency admissions. Frequent health service utilizations imply that people are uncertain about their ability to process health-related information and believe that they lack necessary competences to take care of their health; therefore, they ask for professional help [19].

In order to provide actionable solutions and recommendations for improving the level of health literacy in a society, it is essential to control for demographic and socioeconomic determinants when measuring and assessing the health literacy competencies of a population.

### Implications of limited health literacy

Weak health literacy competencies have several negative consequences, and generally are associated with poorer health outcomes even after controlling for socioeconomic and demographic factors such as income, education, and ethnicity [34]. The WHO also states that limited health literacy is often associated with “*poorer health, less self-management and more hospitalization*” [2]. Limited health literacy significantly impacts a person’s well-being. In addition to deteriorating the quality of life it also has strong negative economic implications for the individuals and the society [2]. The links between patient health literacy and health outcomes, as conceptualised by Lee et al. [35], include three pathways: disease and self-care knowledge; health risk behaviour, preventive care and routine physician visits; and compliance with medication. Individuals with lower health literacy tend to have less knowledge, can be characterized by riskier health behaviour, they take preventive measures irregularly, and often do not comply with medications. As a result, their health status is negatively affected. In addition, they tend to seek assistance with a delay which increases the number of avoidable emergency care visits and hospitalisations.

Empirically, the findings of Lee et al. [35] are supported by a couple of studies [2, 29, 36]. Firstly, people with an inadequate health literacy level are less informed about their health status, less knowledgeable about disease prevention methods and less experienced in self-care practices [29]. Due to their weak health literacy competencies, individuals take their medicine inconsistently and are not able to correctly interpret labels from prescribed medicine [36]. Additionally, as noted by the AMA Foundation and the WHO, people with limited health literacy tend to follow riskier behaviour and make less healthy choices [2, 29]. There is a high probability that these people smoke, eat junk food, take drugs, have insufficient physical activity, and drink alcohol more often – choices which negatively affect their health condition.

According to the AMA Foundation, limited health literacy level leads to *additional costs* of around $60 billion per year in the US due to more hospital days, frequent hospitalizations, and increased workload for emergency departments [29]. This shows that individuals with a lower level of health literacy use treatment services (i.e., having a treatment after being diagnosed with an illness) more frequently than preventive services (e.g., regular check-ups and health screenings), which entails higher costs [37]. Similar results also indicate that by increasing the level of health literacy the usage of hospital and emergency rooms for older people is reduced [38]. Limited health literacy among elderly might be even more dangerous since it can worsen their health condition, which in turn can lead to increased mortality rate [36].

*Non-communicable diseases* such as cancer, diabetes, asthma, heart disease and other chronic diseases are the leading cause of death; according to WHO, in 2016 they accounted for seven of ten deaths worldwide [39]. The effective management of these diseases are dependent on patient health literacy. For example, previous literature documented that people with low health literacy levels suffering from hypertension or diabetes do not possess adequate amounts of information that would allow them to manage their disease in an appropriate way [40]. Additionally, diabetes patients with limited health literacy evaluated the communication of their specialists in the domains of general clarity, description of the condition, and explanation of the care process worse than the patients having a higher level of health literacy [34]. Another study examined the relationship between inadequate health literacy level and chronic disease management [41]. The study revealed that patients with limited health literacy were lacking some basic knowledge about their diseases. The authors documented that only one third of the patients with asthma were aware that it is necessary to regularly check their health condition regardless of having an asthma attack, while 80% of people with adequate health literacy level were well-informed about this aspect [41]. Moreover, people having a low level of health literacy and suffering from asthma were more unwilling to be involved in the decision-making process about the treatment of their disease; they relied on their physicians or family members to make decisions related to their disease [34]. All in all, people suffering from chronic diseases have higher mortality rates if their disease is managed inappropriately [2]. Although approaches to chronic disease management and treatment is rapidly developing, it has become more challenging to interpret medical and dietary instructions, especially for people with limited health literacy [41].

While health literacy plays a crucial role in the prevention and management of non-communicable diseases due to the nature of the illnesses, such competences are also critical in *preventing the spread of communicable diseases*. In particular, the COVID-19 pandemic emerged rapidly along with “infodemic” – abundance of valid and invalid information [42]. In such a situation the ability to comprehend health related information and distinguish truth from misinformation is essential for individual and public safety. For example, Okan et al. [42] showed for the German population that people with higher health literacy could understand coronavirus related information significantly better, while confusion was higher for individuals with lower health literacy skills. Health literacy as a key to quick and efficient response to health emergency situations such as COVID-19 pandemic, has been empathised by several researchers [43, 44]. Until now no empirical study has shown that the level of health literacy is a significant determinant of actual infection. Nevertheless, it is proved that lower literacy is associated with riskier health behaviour, hence we can hypothesize that it might be linked to higher incidence rate.

## Methods

We apply a cross-sectional study design when investigating the level of health literacy and its determinants in Latvia and Lithuania. We employ a mixed research approach – a population-based survey is combined with in-depth interviews with health industry experts. The main aim of the survey is to measure the level of health literacy in Latvia and Lithuania and to find the main drivers influencing its level, while the purpose of the interviews is to gain in-depth insights into the determinants of health literacy and ways to improve health literacy level in Latvia and Lithuania.

### The European health literacy survey

The European Health Literacy Questionnaire developed by Sørensen et al. [9] and abbreviated as HLS-EU-Q is employed in this research. In this way we use questionnaire which has already been validated (pre-tested for face validity in three countries, field-tested with computer-assisted face-to-face interviews in two countries, followed by a consensus-based item selection process) [1]. The survey is divided into three domains: health care, disease prevention, and health promotion. Moreover, it covers four information processing stages: access, understand, apply, and appraise information relevant to health [3, 9]. The final version of the 47-item questionnaire is represented by a matrix and includes twelve sub-dimensions, three domains combined with the four information processing stages, see Table 2 [3, 9, 24].

**Table 2:** Matrix of general health literacy sub-dimensions

Health Literacy	Access/ obtain information relevant to health	Understand information relevant to health	Appraise/ judge/ evaluate information relevant to health	Apply/ use information relevant to health
**Health Care**	1) Ability to access information on medical or clinical issues	2) Ability to understand medical information and derive meaning	3) Ability to interpret and evaluate medical information	4) Ability to make informed decisions on medical issues
**Disease Prevention**	5) Ability to access information on risk factors	6) Ability to understand information on risk factors and derive meaning	7) Ability to interpret and evaluate information on risk factors	8) Ability to judge the relevance of the information on risk factors
**Health Promotion**	9) Ability to update oneself on health issues	10) Ability to understand health related information and derive meaning	11) Ability to interpret and evaluate information on health related issues	12) Ability to form a reflected opinion on health issues

*Source*: HLS-EU Consortium [3], p. 8, Table 1

The HLS-EU Consortium used this matrix for questionnaire construction. The matrix is based on the HLS-EU Conceptual Model [9]

In the survey, participants rate each question on a Likert scale that consists of four anchors: very difficult, fairly difficult, fairly easy, and very easy [1]. This kind of operationalisation allows the subjective assessments of health literacy, and reflets the relational nature of health literacy. The questionnaire serves as an effective tool to measure the respondents’ individual capabilities as well as social competences in relation to health literacy [1].

The results of the HLS-EU-Q survey are transformed into an index that ranges from 0 to 50 – the higher the score, the higher the level of health literacy [3]. The formula for the index calculation, as described in [3], is as follows:

1 $$Index=\left( mean-1\right)\ast \left(\frac{50}{3}\right)$$

where:

*index* is the calculated index of health literacy level;

*mean is* the calculated mean for all the elements considered for each person;

*1* is the lowest value for the mean (transformation required for the index to reach its minimum);

*3* is the range of the mean;

*50 is* the maximum value of the index.

The HLS-EU Consortium defines four levels of health literacy: *inadequate* if the index ranges from 0 to 25, *problematic*, if the index ranges from 26 to 33, *sufficient* if the index is from 34 to 42, and *excellent* if it is 43 or higher [3].

It is important to note that the HLS-EU-Q survey was carefully developed and validated [45]. Most importantly, the development of the HLS-EU-Q followed a concept validation approach; the face validity of the draft questionnaire was tested by focus groups in three countries; and the questionnaire was field-tested in two countries [45]. In addition to the field tests, consultations were organised with experts to assess the construct validity as well as the technical qualities (e.g., scaling, ordering of items) of the questionnaire. The results of the pre-test, field test and expert consultations were pooled and evaluated by experts from the HLS-EU Consortium. The pre-final questionnaire was examined for plain language by literacy experts from Ireland. After its initial usage by the Consortium, the HLS-EU questionnaire became a widely used health literacy survey instrument; it was validated for the general public in several other countries, ranging Portugal in Europe [46] to Indonesia, Kazakhstan, Malaysia, Myanmar, Taiwan, and Vietnam in Asia [47].

For the purpose of this study, the European Health Literacy Questionnaire was translated into three languages - Latvian, Lithuanian, and Russian. The respondents could use the preferred language to answer the questions. As the HLS-EU-Q instrument is used for the first time in Baltic countries, we adapted the questionnaire in Latvian, Lithuanian and Russian languages. We followed the principles as advised by Saboga-Nunes et al. [46] for HLS-EU-Q instrument when using the questionnaire in Portugal. In particular, the questionnaire was translated from English language into Latvian, Lithuanian and Russian, using the TRAPD (Translation, Review, Adjudication, Pretesting, and Documentation) team translation technique, as also recommended by Harkness [48]. The instrument was translated, reviewed, and assessed by three different researchers, the process was followed by piloting all language versions, and finally re-assessed by a native speaker. The translation process employed by the HLS-EU Consortium [45] shall be considered similar to the TRAPD (Translation, Review, Adjudication, Pretesting, and Documentation) team translation technique used in this study.

In order to assess the reliability and internal consistency of the responses, we use the Cronbach’s alpha test, which is considered to be a measure of scale reliability, for further details see Section 2.4 on data analysis. This approach is in line with recommendations and approach used in other medical surveys (e.g., [49–51]).

To comply with Article 6(1) of the General Data Protection Regulation of the EU, we required the participants to confirm their voluntary participation in the survey and give permission to process their answers. The questionnaires used in this study are available as electronic supplementary material (S1).

### Demographic and socioeconomic determinants

In order to understand which factors are associated with the health literacy level in Latvia and Lithuania, it is crucial to include in the questionnaire those specific determinants that are found relevant in previous research. Therefore, in addition to the 47-item HLS-EU-Q questionnaire, questions on the participants’ demographic and socioeconomic status are added. The first determinant included is *gender*. As it has been argued in the literature, gender might be one of the explanatory variables for the level of health literacy. In line with previous empirical evidence, we expect women to have a higher level of health literacy than men [1, 22–24]. Moreover, the *age* of the interviewees is also taken into account as it is a widely used determinant to explain health literacy level [1, 2, 19, 27]. We anticipate a negative relationship between age and health literacy competencies due to deteriorating cognitive functions during the lifetime [27]. Another variable included in the analysis is the *educational level* of the respondents [2, 3, 19, 25–27]. More educated people are expected to make more informed health decisions since they can process and assess health-related information more accurately. In line with the International Standard Classification of Education (ISCED) education levels are divided into six categories (from 0 - pre-primary to 6 - second stage of tertiary education).

As *occupational status* is found to affect health literacy level as well, it is included in the questionnaire (full-time employed, part-time employed, unemployed, retired, other) [3, 29]. As income may also help explaining the level of health literacy [1, 25, 27, 30], the self-perceived *financial situation* is included in the analysis of health literacy determinants as a proxy for income level. Nevertheless, income is a sensitive topic that could lead to lower response rate [52]. As a result, instead of asking the respondents about their annual income, we asked them to rate their financial situation from “very poor” to “excellent”. As *social status* might be another determinant of the level of health literacy [3, 19], respondents are asked to rate their status from “very low” to “very high”. Based on previous research, we anticipate that employment status, income, and social status all have a positive effect on the level of health literacy.

Finally, *frequency of health services usage* is considered in the analysis due to its potential significance in explaining the health literacy level. In the survey, participants shall disclose how frequently they used health services in the last 12 months (0 times, 1 – 4 times, 5 – 8 times, 9 times and more). Based on previous literature we expect a negative relationship between health literacy and the frequency of health service usage [1, 19, 32, 33].

### Sampling and procedures

When measuring the level of health literacy in Latvia and Lithuania, drawing a representative sample is essential for getting valid results. To achieve that we apply stratified random sampling with quota elements.

The stratified random sampling implies dividing the whole population of a country into smaller groups called strata. Afterwards, a certain number of people are randomly chosen from each stratum proportional to the population in the selected area [53]. The quota sampling method is a non-probability sampling technique, which assures that important population characteristics are represented in a sample [54]. Similar technique was employed for country-level analysis in Latvia by several authors [53, 55, 56]. The quota element in the context of this study indicates that the strata are purposefully rather than randomly selected, whereas within the selected strata a systematic random sampling is used.

The target sample size was calculated considering the planned data analysis methods and resources available for the fieldwork. We planned face-to-face interviews to be performed by the research team, not outsourced, hence we made the decision to interview a minimum required sample using random sampling and considering the acceptable level of accuracy. With random sampling, the size of the sample does not have to be related to population size [57]. As a result, we decided to aim for a 95% confidence level and were ready to accept a 10 % margin of error. These requirements yielded the minimum sample size of 97 respondents per country when using the sample size formula of Rea and Parker [58]. Note that the sampling ratio in this study is larger than the sampling ratio for several countries (e.g., Germany, Poland, Spain) in the European Health Literacy Survey [1].

To draw a random sample from Lithuania, Kaunas and its surroundings are chosen as a representative region for the whole country, similarly to other population-based studies from Lithuania [59, 60]. Kaunas can be considered as a region representative of Lithuania due to its central location, size (the second largest city in Lithuania), and the homogeneity between the region’s residents. We requested a random list of 700 addresses from Kaunas and its surroundings; the list was prepared by the State Enterprise Centre of Registers (Valstybės įmonė Registrų centras).

Unlike Lithuania, it is not possible to select only one representative strata for Latvia because the population is not homogenous in terms of education, ethnicity and social status across the country. From the six regions in Latvia, populations in the central part - Riga and Pieriga - can be considered similar to each other; they stand out against other regions in terms of having a population with higher level of education [61], economic activity and employment [62], and more multinationals, including one third of the Russian population [63]. Therefore, in the sample Riga city is representing Riga and Pieriga regions. Populations in Vidzeme, Zemgale and Kurzeme can be assumed to be alike by demographics of education [61], economic activity [62] and ethnicity (higher proportion of Latvian-speaking population than in other regions [63], hence cities Valmiera and Jelgava are representing the regions of Vidzeme, Zemgale, and Kurzeme. Population in Latgale differs from other regions in Latvia by education [61], age (with average age exceeding the country-level average by 2 years) [64], ethnicity (higher proportion of Russian-speaking population) [63], and especially unemployment that exceeds the country-level average by two times [62]. Therefore, Daugavpils city and its surrounding are chosen to represent the region of Latgale. The respective cities were chosen as they are the largest in the area. Therefore, a random sample could be drawn from a larger group of people. In Latvia, a random list of 500 addresses was drawn from the National Address Register [65]. The number of respondents to be interviewed in each location was calculated proportional to the population in the particular region, taking into account the rural-urban split.

As a classical stratified random sampling with quota elements is performed, for analysis purposes the data was weighted to adjust for country-specific demographics for gender and distribution between rural and urban populations.

Face-to-face paper assisted personal interview (PAPI) method has been chosen over online surveying assuring that all population groups are included in the sample frame. In Lithuania, from the random list of addresses obtained from the registers, the interviewer selected every third address up to the point of reaching the target sample size. In Latvia, the random list of addresses was first grouped by sampling locations. Afterwards, the interviewer selected every third address from each sampling location up to the point of reaching the target sample size for that location. If nobody was at home from the household or there was a refusal by the householder to complete the questionnaire, the interviewer selected a new address closest to the designated location. In line with the suggestion outlined in [66], eligible participant were adults aged 18 years or older. Interviews were conducted between 1 December 2019 and 21 January 2020. Interviews were led by Valērija Verdiņa in Latvia, and Ieva Gatulytė in Lithuania. Interviews were typically conducted outside working hours to avoid sampling biases.

### Data analysis

In order to check the reliability and internal consistency of the gathered responses, the *Cronbach’s alpha coefficient* is calculated. The Cronbach’s alpha helps to understand whether the survey items (i.e., questions) correspond to the same dimension [67]. The Cronbach’s alpha fluctuates from 0 to 1, where higher coefficient implies higher reliability of the responses. The level of 0.5 is considered as sufficient to prove internal conformity of the data [67].

We use *Spearman correlation* for identifying statistical dependency and estimating the relationship between the health literacy level and the determinants, following the approach employed by the HLS-EU Consortium [3]. The Spearman correlation is used when data are represented in scales (i.e., Likert scale). This coefficient allows to measure the correlation for non-continuous data [68]. The method transforms scales into ranks; therefore, the data do not have to follow normal bivariate distribution as for the Pearson correlation. Nevertheless, to measure the Spearman correlation coefficient, the relationship between the two variables should be monotonic [68].

In order to measure which determinants are associated with the health literacy level, we apply a *multiple regression model* [3]. The variance inflation factor (VIF) test is employed to check for the multicollinearity of the variables. To fully understand the determinants of health literacy, we run four model specifications. The basic equation is shown in Eq. 2. The variables in the equation are described in Table 3.

2 $${GENHL}_i=\upalpha +{\upbeta}_1{gender}_i+{\upbeta}_2{age}_i+{\upbeta}_3{education}_i+{\upbeta}_4{employment}_i +{\upbeta}_5{finance}_i+{\upbeta}_6{socialstatus}_i+{\upbeta}_7{frequency}_i+{\upbeta}_8{language}_i+{\upvarepsilon}_i$$

**Table 3:** Description of the variables used in the model

Variable	Description
*GENHL*	General health literacy level.
*gender*	Dummy variable for gender: male = 1, female = 0.
*age*	The age groups are as follows: 18-25, 26-35, 36-45, 46-55, 56-65, 66-75, 76 and older.
*education*	The education levels are as follows: ISCED 0: pre-primary education; ISCED 1: primary education; ISCED 2: lower secondary education; ISCED 3: upper secondary education; ISCED 4: post secondary non-tertiary education; ISCED 5: first stage of tertiary education; ISCED 6: second stage of tertiary education [69].
*employment*	Dummy variable for employment status: full-time, part-time, unemployed, retired, other.
*finance*	Financial situation measured by Likert scale from very poor to excellent (very poor, below average, average, above average, excellent).
*social status*	Social status measured by Likert scale from very low to very high (very low, low, lower middle, middle, upper middle, high, very high).
*frequency*	Frequency of health service usage: 0 times, 1-4 times, 5-8 times, 9 times and more.
*language*	Dummy variable for the language used in the questionnaire: Latvian, Lithuanian or Russian.

In three additional specifications, we add dummies for age, education, and frequency of health service usage one by one to the model to understand their specific effect. Each model specification is run three times: for Latvia only, for Lithuania only, and then for both countries together.

### Semi-structured interviews

We perform six in-depth semi-structured interviews with healthcare professionals. We interview a diverse group of experts from the healthcare industry: family doctors, pharmacists and other professionals covering both the public and the private sector. The list of experts and their qualifications are shown in Table 4.

**Table 4:** List of experts interviewed in the study

Name of the interviewee	Country	Date of the interview	Expertise and background
Anna Čukule	Latvia	03/03/2020	Head of Operations and Vice CFO in medical company ARS; more than a 15-year experience in the hospital and healthcare industry. EMBA graduate from SSE Riga.
Pēteris Apinis	Latvia	28/02/2020	Former Minister of Health in Latvia (1995). One of the founders and former president of the Latvian Medical Association (1991-1992 and 2006-2008). Famous doctor and politician in Latvia, honorary doctor of the Latvian Academy of Science, chief editor of the journal Latvijas Ārsts (Latvian Doctor).
Iveta Grigaļūne	Latvia	27/02/2020	Family doctor with more than 20 years of experience.
Aušrinė Armonaitė	Lithuania	28/02/2020	Minister of the Economy and Innovation of Lithuania (2020 -2024). Member of the Parliament of Lithuania (2016-current); member of the Committee on Health Affairs; member of the Parliamentary Group for Development Cooperation and Reproductive Health and Rights.
Raminta Meidutė	Lithuania	29/02/2020	Family doctor with more than 20 years of experience. She is affiliated with the Vilnius University Hospital Santaros Klinikos, one of the most prestigious university hospitals in Lithuania.
Jurgita Slabadaitė	Lithuania	08/03/202	Pharmacy manager at Eurovaistinė (in LT) and EuroAptieka (in LV), the largest pharmacy chain in the Baltic States; extensive experience with patient requests – communication with patients on a daily basis.

The interviews are organized after the survey. With the experts we discuss how they perceive the level of health literacy in Latvia and Lithuania, what can be considered as the major problems, as well as how the current level of health literacy might be improved (e.g., what policies should be introduced).

## Results

### Reliability testing and outliers

The Cronbach’s alpha coefficient is used to validate the internal consistency of the survey responses. The reliability coefficient is estimated for the determinants of health literacy, the level of general health literacy, and the three domains of health literacy (health care, disease prevention, and health promotion). The Cronbach’s alpha shows that the survey is highly reliable when measuring the level of general health literacy (α > 0.94) in the samples drawn from both Latvia and Lithuania. The survey can also be considered as reliable when assessing health literacy in the three domains: health care (α > 0.87), disease prevention (α > 0.86), and health promotion (α > 0.90). For health literacy determinants, the Cronbach’s alpha is 0.50 in Latvia, and 0.64 in Lithuania. Although these coefficients fall outsize the suggested 0.70-0.95 range [49], they still might be considered as acceptable [67].

One response was manually removed from the analysis due to its outlier status. In the Latvian sample, we detected a case of straight lining, providing consecutive identical responses. The respondent, a Latvian male over 76 years of age, marked all statements in the Likert questions as very difficult. We have treated this respondent as an outlier and removed it from the sample [57].

### Sample characteristics

Face-to-face paper assisted personal interviews (PAPI) were conducted with 102 individuals in Latvia and 96 individuals in Lithuania. Table 5a shows the sample distribution between the urban and rural areas in Latvia and Lithuania, while Table 5b shows the gender composition of the sample. The average response rate in the urban area was 48% and 45% in Latvia and Lithuania, respectively. The lowest response rate was documented in Latvia in Jelgava (30%), while the highest in Valmiera (65%). The response rates were typically lower in the rural areas. In the rural areas, on average, only one out of three people were ready to participate in the survey.

**Table 5:** Regional and gender distribution of the sample in Latvia and Lithuania

	Table 5a			Table 5b		
	Urban area	Rural area	Total	Male	Female	Total
*Lithuania*			***96***			***96***
Kaunas	66	30	96	40	56	96
*Latvia*			***102***			***102***
Riga	37	–	37	21	16	37
Valmiera	11	10	21	7	14	21
Jelgava	13	13	26	13	13	26
Daugavpils	9	9	18	9	9	18

**Fig. 1:**
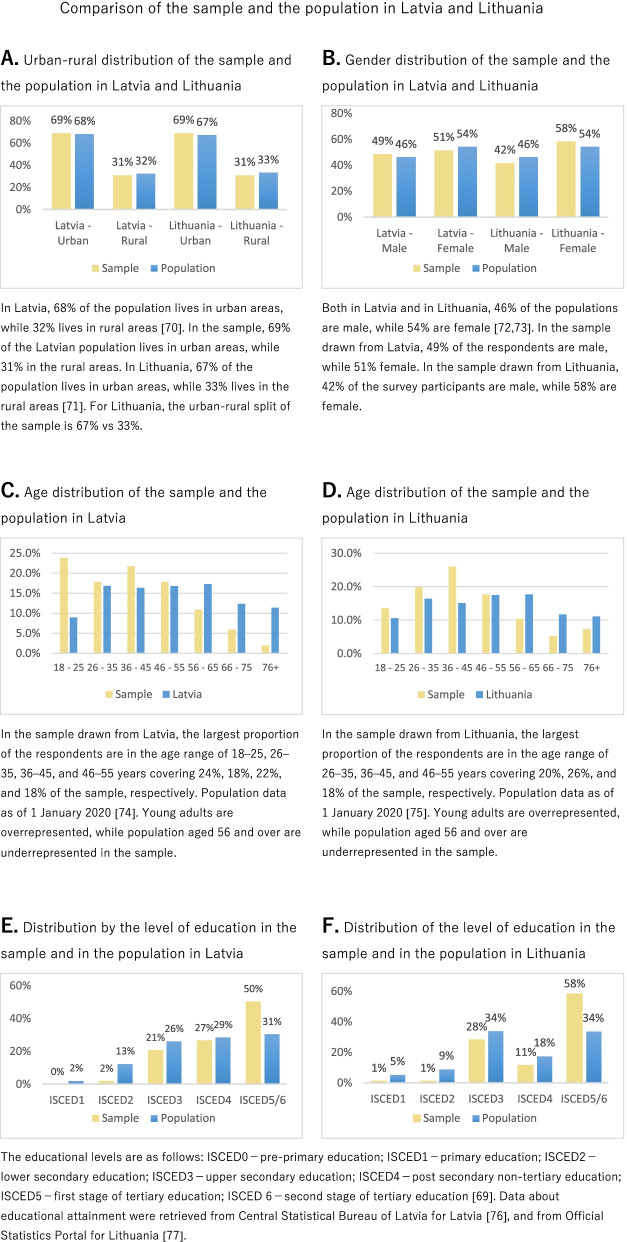
Comparison of the sample and the population in Latvia and Lithuania. **A** Urban-rural distribution of the sample and the population in Latvia and Lithuania [70, 71]. **B** Gender distribution of the sample and the population in Latvia and Lithuania [72, 73]. **C** Age distribution of the sample and the population in Latvia [74]. **D** Age distribution of the sample and the population in Lithuania [75]. **E** Distribution by the level of education in the sample and in the population in Latvia. **F** Distribution of the level of education in the sample and in the population in Lithuania [69, 76, 77]

The sampling technique (stratification) ensured that the sample is representative of the population with respect to two chosen population parameters: urban-rural distribution (Fig. 1A) and gender composition (Fig. 1B). The age distribution of the survey participants is similar in the two subsamples. In the subsamples, younger people are overrepresented (aged 45 and younger) and older people (aged 65 and older) are underrepresented when compared to the age distribution of the population of the respective countries (Fig. 1C and D). Regarding the level of education, in both subsamples, individuals having higher education are overrepresented, while individuals having lower secondary education are underrepresented when compared to the educational attainment of the population of the respective countries (Fig. 1E and F).

**Fig. 2:**
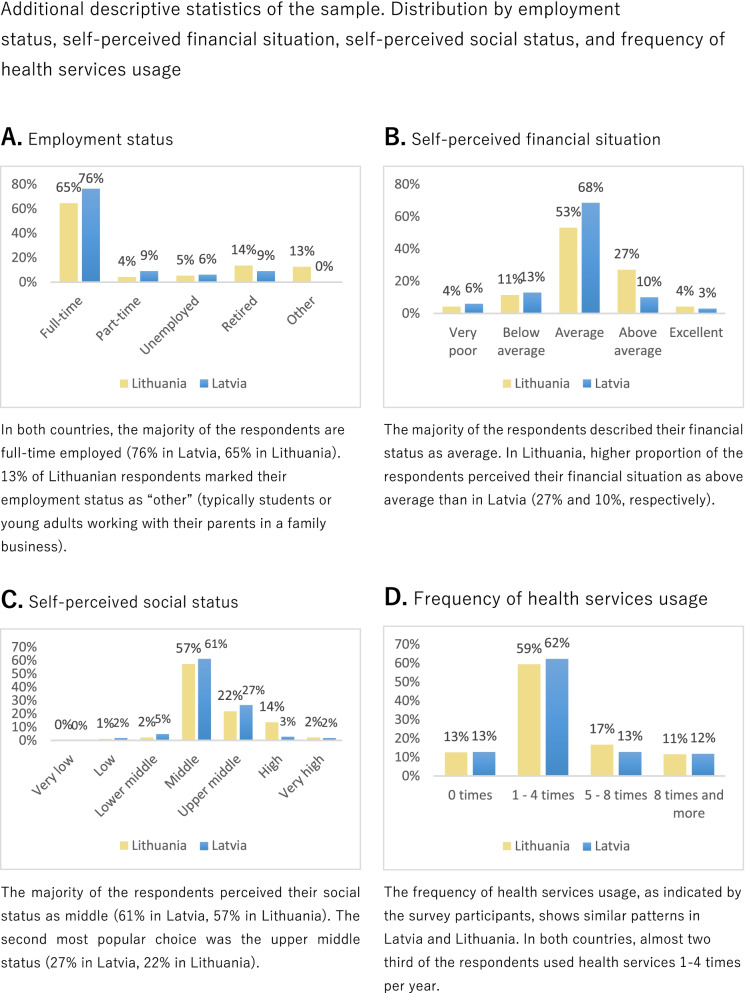
Additional descriptive statistics of the sample. Distribution by employment status, self-perceived financial situation, self-perceived social status, and frequency of health services usage. **A** Employment status. In both countries, the majority of the respondents are fulltime employed (76% in Latvia, 65% in Lithuania). 13% of Lithuanian respondents marked their employment status as “other” (typically students or young adults working with their parents in a family business). **B** Self-perceived financial situation. The majority of the respondents described their financial status as average. In Lithuania, higher proportion of the respondents perceived their financial situation as above average than in Latvia (27% and 10%, respectively). **C** Self-perceived social status. The majority of the respondents perceived their social status as middle (61% in Latvia, 57% in Lithuania). The second most popular choice was the upper middle status (27% in Latvia, 22% in Lithuania). **D** Frequency of health services usage. The frequency of health services usage, as indicated by the survey participants, shows similar patterns in Latvia and Lithuania. In both countries, almost two third of the respondents used health services 1-4 times per year

Additional descriptive statistics of the sample are presented in Fig. 2A-D. Figure 2A shows the distribution of the survey participants by employment status, while Fig. 2B presents the sample composition in terms of self-perceived financial situation. Figure 2C shows the distribution of the survey participants by social status, while Fig. 2D provides information about the frequency of health services usage of the respondents.

### Levels of health literacy in Latvia and Lithuania

From the survey results the general health literacy levels in Latvia and Lithuania were derived following the methodology developed by the HLS-EU Consortium [3]. In Latvia, the value of the HLS-EU-Q index as described in Eq. 1 is 28.46. In Lithuania, the value of the HLS-EU-Q index is 29.89. As the value of the indices fall within the range of 26-33 in both countries, the level of health literacy can be considered problematic both in Latvia and Lithuania [3]. Electronic Supplementary Material S2 displays the distribution of the responses to the 47 survey questions of the European Health Literacy Questionnaire.

**Fig. 3:**
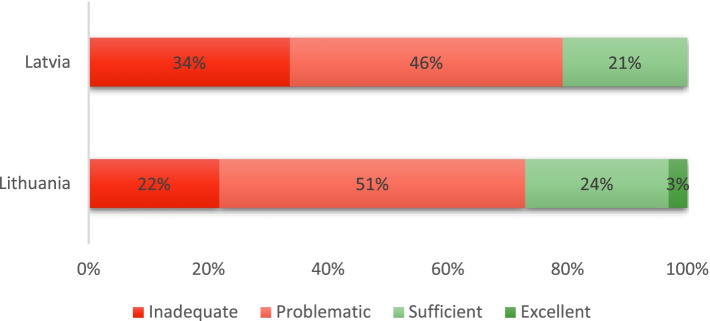
Levels of general health literacy in Latvia and Lithuania. The distribution of general health literacy levels in Latvia and Lithuania using the 47-item European Health Literacy Questionnaire. The general health literacy levels are calculated following the index calculation methodology developed by the HLS-EU Consortium [3]. The level of health literacy is inadequate if the index ranges from 0 to 25, problematic, if the index is from 26 to 33, sufficient if the index ranges from 34 to 42, and excellent if it is 43 or higher [3]

Figure 3 segments the respondents into four clusters according to their general health literacy level. In Latvia, 34% of the respondents have an inadequate health literacy level. The same figure is 22% for Lithuania. The proportion of individuals with problematic general health literacy level is higher in Lithuania than in Latvia (51% vs 46%). Considering inadequate and problematic levels as manifestations of limited health literacy, 79% of the population in Latvia, while 73% of the population in Lithuania may face problems resulting from limited health literacy. At the same time, 21% of the respondents have a sufficient level of general health literacy in Latvia; the same figure is slightly higher in Lithuania (24%). In Lithuania, 3% of the respondents can be characterized by an excellent health literacy level. In Latvia, no survey participant achieved that level (Fig. 3).

**Fig. 4:**
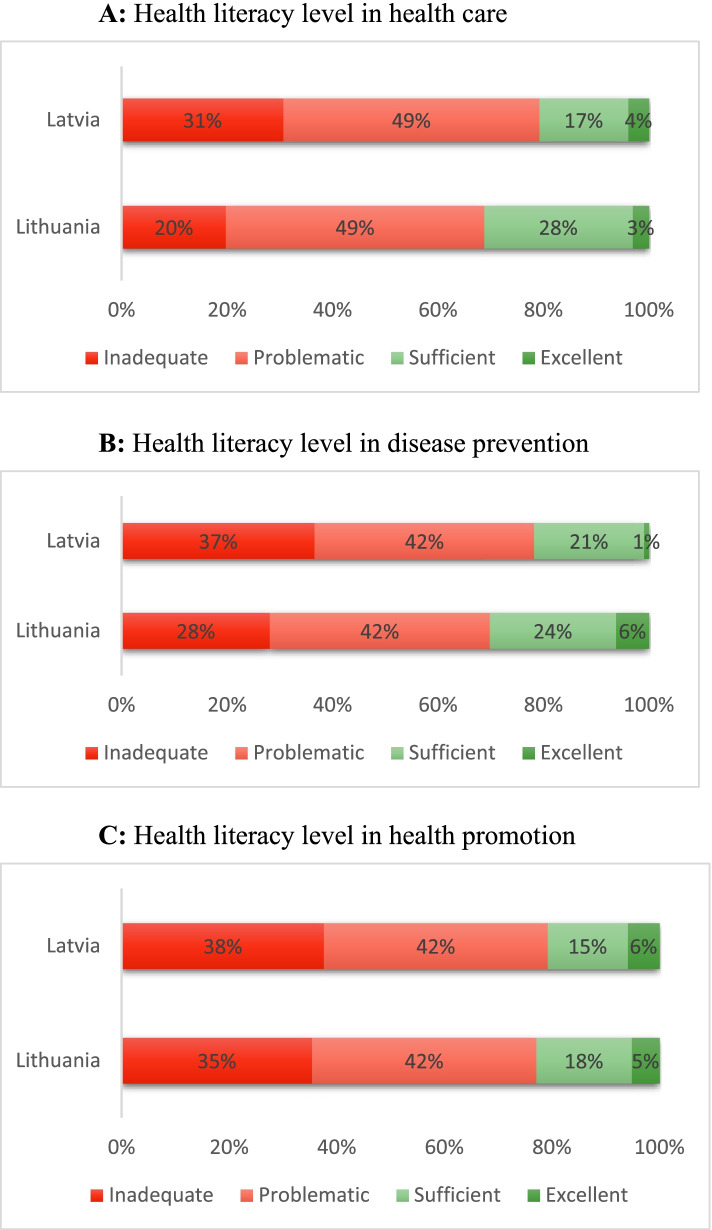
Levels of health literacy in three domains (healthcare, disease prevention, and health promotion) in Latvia and Lithuania. **A** Health literacy level in health care. **B** Health literacy level in disease prevention. **C** Health literacy level in health promotion. The figures show the distribution of the health literacy levels in three domains in Latvia and Lithuania using the 47-item European Health Literacy Questionnaire. From the 47 items 16 items assess the individuals’ ability to access, understand, apply, and appraise information relevant to health in health care, 15 items are related to disease prevention, while 16 items assess the health literacy of individuals in the domain of health promotion. The health literacy levels in each domain are calculated following the index calculation methodology developed by the HLS-EU Consortium [3]

In addition to the level of general health literacy, the European Health Literacy Survey allows to evaluate the levels of health literacy in three domains (health care, disease prevention, and health promotion), see Fig. 4A-C. The highest literacy level is obtained in the health care domain with the value of the index being 29.08 and 31.3 in Latvia and Lithuania, respectively. The lowest literacy level is detected in the disease prevention domain in Latvia (28.01), and in the health promotion domain in Lithuania (28.56). Limited health literacy (inadequate and problematic levels) is around 78-79% in Latvia in all three domains, while it fluctuates between 69 and 73% in Lithuania.

### Determinants of health literacy in Latvia and Lithuania

#### Correlation analysis

Spearman correlations are calculated to characterize the relationship between the general health literacy level and its determinants. Correlation analysis is performed for Latvia and Lithuania separately, and for the two countries jointly (Table 6). In Latvia, the financial situation and the frequency of health services usage correlates positively with the general health literacy level. In Lithuania, the general level of health literacy correlates negatively with age and the frequency of health services usage, while positive correlations are observed between the general health literacy level and the financial situation and the social status, respectively. In addition, the health literacy level correlates negatively with retirement when compared to full-time employment (Table 6).

**Table 6:** Spearman correlations between determinants and general health literacy level

General Health Literacy	Latvia	Lithuania	Total
Gender	−0.0085	0.0786	0.0278
Age	0.0454	−0.2782***	−0.1079
Education	0.1108	0.0209	0.0684
Part-time employed	0.1329	−0.1054	0.0313
Unemployed	0.0287	−0.0508	−0.0078
Retired	−0.0930	−0.2231**	− 0.1633**
Other employment	–	0.0438	0.0588
Financial situation	0.2130**	0.3191***	0.2829***
Social status	0.1525	0.3109***	0.2480***
Frequency of health services usage	0.1678*	−0.1785*	−0.0023

In the pooled sample, statistically significant positive correlation with the general health literacy level is observed for the financial situation and the social status. In addition, health literacy level and retirement correlates negatively (Table 6).

#### Multiple regressions

Multiple regressions are employed to identify the determinants of the health literacy level (Table 7). The regressions are tested for multicollinearity; variance inflation factor (VIF) test is employed. As all VIF values are lower than 3, multicollinearity is not a problem in these regressions [78]. In particular, the highest VIF value is 2.48, while the second highest is 1.44.

**Table 7:** Results of the multiple regressions (* p < 0.05; ** p < 0.01; *** p < 0.001)

	Latvia				Lithuania				Latvia and Lithuania (pooled sample)			
VARIABLES	Eq.1	Eq.2	Eq.3	Eq.4	Eq.1	Eq.2	Eq.3	Eq.4	Eq.1	Eq.2	Eq.3	Eq.4
Gender	−0.780	−1.020	− 0.510	−0.840	− 0.198	−0.290	0.0846	−0.212	− 0.252	−0.354	− 0.0285	−0.294
Age	0.282		0.353	0.336	−0.476		−0.544	−0.470	− 0.0248		− 0.0329	−0.0176
26 – 35		1.950				−4.509^*^				−1.098		
36 – 45		2.276				−1.875				0.648		
46 – 55		1.216				−4.884^*^				−0.649		
56 – 65		2.682				−2.674				0.263		
66 – 75		1.511				−0.0651				1.781		
76 and older		−3.353				−10.19^**^				−7.155^**^		
Education	−0.0317	−0.429		−0.0685	−1.209	−0.503		−1.216	−0.387	− 0.395		− 0.352
ISCED1							−1.993				−6.349^*^	
ISCED2			−9.956^**^				−6.566^**^				−7.135^**^	
ISCED3			0.410				3.114				1.403	
ISCED4			−0.783				1.940				0.620	
ISCED6			−7.448^***^				−6.977^***^				−4.949^***^	
Part-time	3.132	3.347	2.872	3.125	−4.092	−4.577^*^	−4.040	−4.179	0.629	0.604	0.533	0.604
Unemployed	1.930	1.937	5.330^**^	1.880	−3.707	−3.650	−4.144	−3.722	−0.929	−1.474	0.359	−1.035
Retired	−3.912	−2.298	−4.126	−4.482^*^	−2.311	−0.714	−1.689	−2.293	−2.924	− 1.206	−2.707	− 3.113
Other					− 2.241	− 2.727	− 2.798	−2.282	−0.0995	−0.395	− 0.533	−0.0131
Financial situation	1.242	1.284	1.191	1.226	0.747	1.030	1.013	0.760	0.941	1.134	1.123	0.904
Social status	1.191	1.643	1.090	1.240	2.216^**^	1.431	1.770^*^	2.219^**^	1.736^**^	1.432^**^	1.420^*^	1.740^**^
Frequency of health services usage	1.532^*^	1.632^*^	1.824^**^		−1.634^**^	−1.336*	−1.206		−0.105	0.0649	0.123	
1 – 4 times				0.542				−2.081				−0.702
5 – 8 times				1.682				−3.341				−1.039
9 and more				4.708^*^				−5.255^**^				−0.245
LV									−2.920^**^	−2.888^***^	−2.997^***^	−2.912^**^
RU	2.756^**^	2.744^**^	3.039^**^	2.716^**^					0.545	−0.0878	0.502	0.512
Observations	101	101	101	101	96	96	96	96	197	197	197	197
R-squared	0.171	0.193	0.220	0.177	0.261	0.349	0.297	0.261	0.155	0.207	0.190	0.157

In Eq.1. all variables are included as continuous/categorical variables as appropriate. In Eqs. 2-4 three variables are included as dummy variables. In Eq.2 age is included as a dummy variable (dummies are added to the age groups), while all other variables are the same as in Eq.1. In Eq.3, education is included as a dummy variable (dummies are added to the educational levels), while all other variables are the same as in Eq.1. In Eq.4, the frequency of health services usage is included as a dummy variable (dummies are added to the categories of usage frequency), while all other variables are the same as in Eq.1

For Latvia, the multiple regressions analysis indicate that unemployed individuals have higher health literacy level, while retired people tend to have a lower health literacy level when compared to the population being full-time employed. Although the result for unemployed individuals is counterintuitive, it can be explained by the fact that students also marked themselves as unemployed instead of ticking the “other” employment category (there is no such observation at all). In Latvia, the level of health literacy is positively associated with the frequency of health services usage. This effect is more pronounced for individuals visiting the health care institutions more than nine times a year. Additionally, individuals with lower secondary education (ISCED2) tend to have a lower health literacy level when compared to individuals with higher education (ISCED5). On the contrary, individuals who obtained a doctoral degree (ISCED6) have a lower level of health literacy compared to individuals with a bachelor’s degree (ISCED5). Nevertheless, this result contradicting the literature is based on a single observation in this category (ISCED6). Finally, in each model specified for Latvia the results show that individuals answering the survey questions in Russian tend to have a higher health literacy level than those who filled it out in Latvian (Table 7).

For Lithuania, the results show that social status is a significant determinant of the health literacy level in three out of four model specifications; the higher the social status, the higher the health literacy level. The effect of education on the health literacy level is similar to the one observed in Latvia. When age is added as a discrete variable, age is an insignificant determinant of health literacy. However, when considering each age group separately, the effect is negative for several age groups. In particular, individuals in the age groups of 25-36, 46-55, 66-75 have a lower health literacy level compared to individuals aged between 18 and 25 years. In Lithuania, in contrast to the findings for Latvia, the frequency of health care services usage is negatively associated with the health literacy level. This significant association can be attributed to respondents visiting healthcare institutions more than nine times per year (Table 7).

The last four columns of Table 7 show the results for Latvia and Lithuania jointly. The analysis reveals that social status is one of the strongest determinants of the health literacy level in both countries. Language is also a highly significant determinant of health literacy. In particular, we find that the Latvian-speaking population has a lower health literacy level compared to the Lithuanian-speaking population. In addition, when we add educational levels as separate variables to the regressions, some significant associations can be observed. Individuals with primary (ISCED1) and lower secondary education (ISCED2) can be characterized with a lower health literacy level compared to individuals with a bachelor’s degree (ISCED5). Similarly, a significant negative effect can be observed for individuals with a doctoral degree (ISCED6); a result which might be biased due to the small sample size (*n = 2*). Finally, when age is included as a categorical variable (Model 2), elderly (aged 76 and older) have lower health literacy level compared to the youngest individuals in the sample (aged 18-25).

### Improving the level of health literacy in Latvia and Lithuania

Given the limited level of health literacy in Latvia and Lithuania, it is essential to assess the potential means for improving it. The measures outlined here are based on the expert interviews. Both in Latvia and Lithuania, the first and most important area identified by experts is *health education* in schools; all experts interviewed see a significant gap in this area. Mr. Apinis notes that typically parents transmit health knowledge to their children in early childhood. Nevertheless, if parents lack knowledge in this area, children will not have sufficient knowledge for maintaining their health. Therefore, the only place where these children can obtain relevant information about health is schools. Ms. Čukule, Ms. Grigaļūne, and Mr. Apinis argue that the basic school program should include a separate subject primarily focusing on health education. At the same time, Ms. Maidutė stresses the importance of adapting the educational programmes to meet the needs of young children; the basic concepts should be explained in simple terms applying visualisation and association methods. In addition, Ms. Čukule argues that health education should be promoted not only in schools but also in workplaces.

A second suggestion outlined by the experts is to *improve health-related information transmission* to the general population as well as to improve health promotion among the society. Mr. Apinis argues that the Latvian-speaking population lacks information resources from which they might draw relevant and up-to-date knowledge about health in their native language. Ms. Meidutė, Ms. Slabadaitė, Ms. Grigaļūne and Mr. Apinis point out that even if there is some information available, its unstructured nature discourages people from obtaining and absorbing it. Ms. Grigaļūne adds that several times doctors are unable to follow new regulations in the healthcare industry due to its unstructured form and poor quality of information transmission.

Finally, the experts agree that a *user-friendly database* including health-related information might improve the health literacy levels in Latvia and Lithuania. However, they note that the description of diseases, symptoms and its management might harm people given the limited health literacy of the population. Individuals lacking basic health knowledge will be unable to correctly interpret the information provided. The experts stress that the list of guidelines should be written in a clear and straightforward manner helping the readers to deal with their health problems.

## Discussion

### Levels of health literacy in Latvia and Lithuania

The samples drawn both from Latvia and Lithuania are representative of the whole population in terms of gender, urban/rural distribution and regions. Stratification ensured that the sample is representative in terms of these parameters; due to time and monetary constraints we could not control for additional parameters. In terms of age, the sample is not fully representative of the population of Latvia and Lithuania; elderly (people aged 65 and older) are underrepresented in the sample. In general, elderly were less willing to participate in the survey due to their negative perception towards surveys [79]. As older people typically have lower health literacy levels [2, 19, 27], had we managed to interview more individuals aged over 65, the level of health literacy would be even lower. In terms of education, the sample is also not fully representative of the population; individuals with higher education are oversampled. Most probably, more educated individuals were more willing to participate in the survey understanding its importance. As individuals with higher education are typically more health literate [2, 19, 27], the level of health literacy in Latvia and Lithuania might be even lower than the one reported in this study due to oversampling more educated people.

**Fig. 5:**
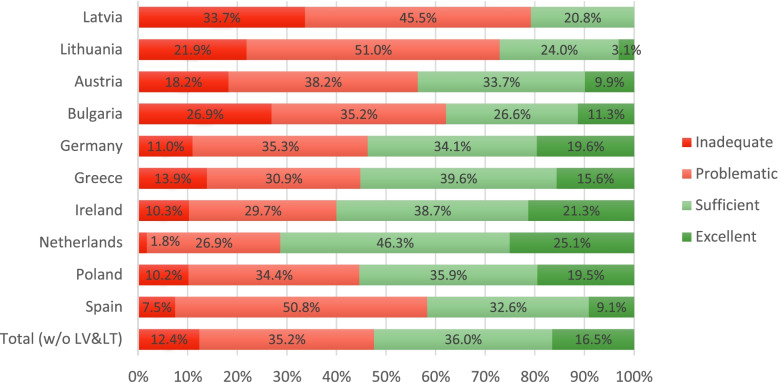
Levels of general health literacy in ten European countries. The distribution of the general health literacy levels in Latvia and Lithuania using the 47-item European Health Literacy Questionnaire. The general health literacy levels are calculated following the index calculation methodology developed by the HLS-EU Consortium [3]. Data for countries other than Latvia and Lithuania were taken from HLS-EU-Consortium [3]

Most importantly, we find that the general health literacy levels in Latvia and Lithuania are lower than the ones documented in eight European countries. According to the HLS-EU Consortium [3], the distribution of general health literacy levels in Europe are as follows: 12% inadequate, 35% problematic, 36% sufficient, and 17% excellent (Fig. 5).

Nevertheless, the proportion of individuals with limited health literacy is much higher in Latvia and Lithuania than in other European countries. Inadequate and problematic health literacy levels account for 79 and 73% of the responses in Latvia and Lithuania, respectively. In addition, the HLS-EU Consortium [3] reported the largest proportion of individuals with inadequate health literacy level for Bulgaria (26.9%), and this proportion is even higher in Latvia (34%) (Fig. 5). All in all, we may conclude that in Latvia and Lithuania, on average, the problem of limited health literacy is more pronounced than in the eight European countries sampled by the HLS-EU Consortium [3].

The limited health literacy of the population in Latvia and Lithuania is reflected in the poor health status of the population and several core health indicators. The life expectancy and the healthy life years at birth are among the lowest in Latvia and Lithuania when compared to other European countries [7]. Death attributable to the main causes of mortality (e.g., ischaemic heart diseases, cerebrovascular diseases, cancer, respiratory diseases) are also among the highest [7]. Similarly, the avoidable (preventable and treatable) mortality rates are among the highest in Latvia and Lithuania when compared to other European countries [7]. Moreover, the avoidable hospital admissions are above the EU average [7]. Most probably, several premature deaths and hospital admissions could have been avoided through higher health literacy. Finally, the low health literacy of the Latvian population is also reflected in the proportion of the population being fully vaccinated against covid among adults. The ratio is the third lowest in Latvia (43.6); the vaccine rate is lower only in Bulgaria and Romania [80].

### Determinants of health literacy

When discussing the determinants of health literacy, we complement the survey results with the insights gained from the expert interviews. According to the survey results, *gender* neither correlates with the health literacy level nor is a significant determinant of it (Tables 6 and 7). This result is in line with the strand of research documenting insignificant association between gender and the health literacy level [3, 25, 26]. Nevertheless, during the interviews Ms. Armonaitė, Ms. Meidutė, Ms. Slabadaitė, and Ms. Čukule argued that gender is a significant driver of health literacy level. They emphasized that women care more about their health leading to higher levels of health literacy; a relationship documented in the literature [1, 18, 22–24]. Ms. Armonaitė provides another valuable insight; she believes that the traditional gender roles are highly influential. In particular, men care about their health less due to the social perception that men are strong and healthy. Therefore, their commitment to regular check-ups is lower when compared to women, they try to avoid vulnerability in this way.

Regarding *age*, the Spearman correlation analysis reveals a negative and significant correlation between age and health literacy level in Lithuania, while this relationship is insignificant in Latvia (Table 6). The multiple regressions reveal that elderly (people aged 76 and older) have a lower health literacy level when compared to the youngest age group in the total sample (Table 7). This finding is in line with previous research documenting that older people are negatively affected by their weak health literacy competencies due to their deteriorating cognitive abilities [1, 2, 19, 27, 28]. Experts share the same opinion; during the interviews Ms. Čukule, Ms. Meidutė and Ms. Slabadaitė emphasize the importance of age while evaluating health literacy level. Ms. Meidutė argues that young people are more receptive to information, they tend to have more experience with innovative information tools, which in turn increases their knowledge.

Empirical evidence shows that *education* has a strong positive influence on the level of health literacy; people with higher education tend to have stronger health literacy competencies [2, 25–27]. In this research, some but not all multiple regression models find support for it (Table 7). From the experts’ point of view, education is the most important determinant of the health literacy level. Ms. Grigaļūne claims that several Latvians lack basic knowledge about their health and body, which could be considered as a critical problem in the country. Most of the patients with whom she interacts as a family doctor, do not know the location and the function of their main organs. Mr. Apinis adds that people with higher education tend to live longer, while Ms. Čukule mentions that educated people tend to be more aware and conscious about their health. All in all, according to the experts’ opinion, education is one of the most important and influential determinants of health literacy level in both countries.

Another determinant of health literacy emphasized by AMA Foundation and HLS-EU Consortium is *employment* [3, 29]. Findings of this study suggest that being retired is negatively associated with the health literacy level in Lithuania and in the pooled sample (Table 7). In contrast, the experts interviewed do not consider employment status as a crucial driver of the health literacy level. Nevertheless, Ms. Slabadaitė adds that people performing intellectual work tend to have a higher level of health literacy.

Previous literature suggests that *income* is positively associated with the health literacy level [1, 2, 25, 27]. In this research, although the Spearman correlations analysis shows a positive correlation between the financial situation and the health literacy level (Table 6), the multiple regressions reveal an insignificant relationship between the two variables (Table 7). The strong correlation between the financial situation and the social status might explain this phenomenon. The experts’ opinion overlaps with the conclusions drawn from the literature and the Spearman correlation analysis. Ms. Grigaļūne, Ms. Čukule, Ms. Armonaitė, and Ms. Slabadaitė share the opinion that the financial situation might partly explain the level of health literacy. Ms. Čukule claims that individuals with higher income typically have better health insurance implying that they have a better access to health services. In addition, individuals with better financial situation have the opportunity to use private medical services which makes the treatment process less stressful and time-consuming. As a result, they use the health services more frequently, while individuals with limited financial resources avoid regular check-ups due to monetary constraints. Mr. Apinis asserts that education is strongly associated with the financial situation; therefore, by improving the level of education the income level would increase which, in turn, would improve the health literacy level.

Additionally, empirical evidence suggests that higher *social status* is associated with higher health literacy level [2, 19]. Similar conclusions can be drawn from this research for Lithuania and for the total sample; the Spearman correlations show a significant positive correlation between the social status and the health literacy level (Table 6). Additionally, the multiple regression model indicate that social status is a significant driver of the health literacy level in Lithuania (Table 7). The expert interviews confirm the above finding. Ms. Grigaļūne, Ms. Čukule, and Ms. Slabadaitė share the opinion that social status is indeed an important determinant of the health literacy level.

In the literature, the frequency of *health services usage* is documented to be negatively associated with the health literacy level [1, 19, 32, 33]. In this research, in line with previous evidence, we find a significant negative relationship between the two variables in Lithuania (Tables 6 and 7). In Latvia, surprisingly, a positive relationship is observed (Tables 6 and 7). In the pooled sample this determinant is insignificant (Tables 6 and 7). Nevertheless, Ms. Grigaļūne, Ms. Čukule, and Ms. Meidutė consider the frequency of health services usage as an important driver of the health literacy level. In particular, Ms. Grigaļūne and Ms. Čukule claim that there indeed shall be a positive association between the frequency of health services usage and the health literacy level in Latvia. They argue that if individuals with limited health literacy visit doctors more often, they acquire new information and become more knowledgeable about their health. In contrast, Ms. Slabadaitė believes that the frequency of health services usage does not influence the health literacy level at all—claim matching the results from the pooled sample.

Finally, the *language* used by the respondents (Latvian, Lithuanian, Russian) shall be considered as an important determinant of the health literacy level. The multiple regressions analysis indicates that individuals answering the survey questions in Russian or Lithuanian have a higher level of health literacy than those who filled out the survey in Latvian (Table 7). Mr. Apinis reasons that limited amount of information is provided to the general population about health in Latvian. Moreover, if some information is available, typically provided by the authorities, it is quite challenging to grasp and interpret it. As a result, the general public does not profit from these information sources. In his opinion, insufficient amount of information is one of the most important factors explaining the limited health literacy in Latvia.

The expert interviews revealed some *additional determinants* not covered in the previous literature and thus not investigated in the survey. First, Ms. Armonaitė mentions that the level of health literacy strongly depends on *access to health service*s. Although some individuals would prefer checking their health more often, there are considerable waiting times. In addition, some specialists have a wrong attitude towards those patients who visit them for a check-up which discourages individuals from doing so. Second, Ms. Slabadaitė mentions the ability to *access health-related information* easily. Nowadays, there is a lot of health-related information on TV, social media, newspapers, and the Internet. The easier the access to this information, the higher the probability of understanding the underlying health condition, which in turn might increase the level of health literacy in the long term. Third, according to Ms. Grigaļūne, *traditions* affect the health literacy of individuals as well. These traditions, passed down from generation to generation, include eating habits and the use of non-prescription drugs. Therefore, parents play a crucial role in affecting their children’s attitude towards health and healthy lifestyle. Finally, Ms. Grigaļūne and Ms. Čukule stress the importance of the *value and priority of health*. They claim that in Latvia health is not valued untill sickness comes. As a result, people are not interested in obtaining health-related information, caring for themselves, and enhancing their health literacy.

### Improving the level of health literacy

The higher the level of health literacy in a population, the higher the benefits for the society [2]. People with strong health literacy competencies tend to be richer, contribute more to the country’s economic development, have a better health condition, and superior quality of life [2]. The improved level of health literacy – through the acquisition of new knowledge – leads to positive attitudes and behaviours, higher self-efficacy, and better health outcomes [81]. Given their better health outcomes, health literate individuals are more productive and make a more active contribution to the social welfare system [4]. On system level, by improving the level of health literacy, the usage of health services declines, and the cost of hospitalization decreases [82, 83].

Given the high benefits of improved health literacy both at individual and system level, policy makers shall commit to increase its level. A detailed study for the US suggested the following measures to improve the level of health literacy [84]: *i)* the curriculum in the education system should include materials, tasks, different cases, and examples related to health from early ages; *ii)* both professional educational institutions and health education programs should include the concept of health literacy in their curriculum; *iii)* healthcare programs reducing the negative implications of limited health literacy shall be created and promoted; and finally, *iv)* the government and private investors shall stimulate various research in the field of health literacy, including its measurement.

Expert interviews suggested that the most important area for improvement is *health education* in schools both in Latvia and Lithuania. All experts interviewed perceived a significant gap in this area. This perception is in line with Nielsen-Bohlman et al. [84], who argue that it is important to start educating people in healthcare from a young age.

Second, experts underlined the importance of *improving health-related information transmission* to the general population as well as *health promotion* among the society. This suggestion is in line with Nielsen-Bohlman et al. [84] who assert that one of the first steps in improving health literacy level in a country is to increase the society’s awareness and knowledge about health-related topics. All things considered, the expert interviews suggested that improving the flow and availability of information in the media (i.e., radio, television, news portals, etc.) might enhance the level of health literacy in both countries.

Third, the experts shared the opinion that a *user-friendly database* incorporating health-related information might enhance the health literacy levels in Latvia and Lithuania. In general, the experts shared the opinion that a database with detailed instructions for the most common health problems (e.g., fever, cough, vomiting, headache, etc.) might help the sick in self-medication and understanding its consequences, while at the same time educating them.

### Limitations and future research

This research has a number of limitations. *First*, although the sample is representative in terms of gender, urban-rural distribution and regions, it is limited (approx. 100 respondents per country). The survey responses were gathered on-site; the interviewers had to travel to several urban and rural sites making the process both time-consuming and costly. Due to the small sample size, in some less frequent categories there are only a few observations. Such less frequent categories include no school education, primary education and doctoral degree when measuring the educational level; very poor and excellent when assessing the financial situation, and very low and very high when measuring the social status. *Second*, when gathering responses, determinants such as gender and age are evident while others come forth only when an individual fills out the questionnaire. It is hard to control for determinants unknown before running the survey (e.g., education, employment, financial situation). As a result, individuals with particular characteristics are oversampled (e.g., young adults, individuals with higher education) while others are undersampled (e.g., elderly, individuals with lower secondary education). Given the limited sample size and the over/under-representation of particular subpopulations, some significant relationships might be hidden while others shall be interpreted with caution. *Third*, we could not assess whether the sample is representative by ethnicity as we did not include any ethnicity-related questions among the socio-demographic characteristics due to its sensitivity and multidimensionality [85, 86]. Hence, the sample might not be representative in terms of ethnicity. *Fourth,* surveying individuals from Kaunas and Kaunas region might bias the results due to the positive externalities related the Lithuanian University of Health Sciences, which is the largest medical training school in Lithuania. Positive externalities include, for example, the medical research projects carried out in the region and the high number of practicing physicians when compared to other regions of Lithuania. As a result, the level of health literacy in the population might be lower than the one estimated in the study.

Moreover, the research could be subject to a number of biases. *Recall bias* refers to a situation when a person has difficulties in recalling a notable event and, therefore, fails to report it correctly [87]. In this research, recall bias is present as respondents might not be able to remember correctly how many times they used health services during the last 12 months. The *social desirability bias* – tendency to respond in a socially acceptable or desirable manner – may be present. In the questionnaire respondents are asked to assess to what extent they find it difficult or easy to perform different health related activities, and they may feel tempted to look more competent and answer that certain functions are easier than in reality. *Conformity bias* implies that individuals follow the behaviour of a group of people instead of revealing their own judgment [88]. This bias might explain why the majority of the respondents rated their financial situation and social status as average even if their socioeconomic status is above/beyond the average. As some people refused to participate, *non-response bias* might be present. Typically, older people were less willing to participate in the survey. On the one hand, they were rejective due to perceiving the interviewers as salespersons with the ultimate aim of selling a product or service at the end. On the other hand, the questionnaire seemed too long and complicated for them. For example, there were some elderly who started to fill out the questionnaire but refused to finish after realizing how challenging and time-consuming it is. The non-response bias resulted in under-sampling elderly whose health literacy competencies are typically weak. Moreover, individuals with insufficient health related knowledge might have also refused participating in the survey due to their discomfort, again leading to the underestimation of the general health literacy level. Other than the biases listed in this paragraph, we do not expect the study to suffer from survey biases. Most importantly, we employed a validated and widely used questionnaire (HLS-EU-Q) developed by the HLS-EU Consortium providing the benefit of comparing results across various countries.

*Future research* might aim at increasing the sample size. A larger sample size would enhance the sample’s representativeness by obtaining more observations in the less frequent categories (e.g., individuals with lower secondary education or with a doctoral degree). Evidently, the larger the size of a representative sample, the more valid the results are. A larger sample nevertheless would require additional resources for hiring and training interviewers. Future research might also investigate the association between the level of health literacy and some additional determinants. There are a couple of determinants which were considered as important by the experts but not covered in the previous literature (e.g., access to health services, traditions). The inclusion of these determinants into the survey would lower the probability of having omitted variable bias. Finally, to gain a deeper insight into the most promising ways of improving the health literacy levels in Latvia and Lithuania, interviews with additional healthcare experts could be conducted. Such interviews, as suggested by Mr. Apinis, might follow the Delphi method which would help to form a group opinion about the most efficient ways of promoting the level of health literacy.

## Conclusions

Although the level of health literacy has already been assessed in a few European countries, it has never been measured in Latvia and Lithuania. In this research, we investigated the level of health literacy and the factors associated with it in these two Baltic countries. In doing so, we employed a validated questionnaire developed by Sørensen et al. [9]. We conducted face-to-face paper-assisted surveys in various urban and rural areas in Latvia and Lithuania. Although the sample is small, (approx.100 responses were recorded in each country), it is representative allowing us to draw reliable conclusions. Furthermore, in order to assess potential means for improving the level of health literacy, in-depth interviews were conducted with various experts from the healthcare industry. During these interviews, the experts provided suggestions for improving the limited level of health literacy.

Most importantly, the 47-item European Health Literacy Questionnaire (HLS-EU-Q) conducted among the population in Latvia and Lithuania revealed that the health literacy level is problematic in both Baltic countries. The survey results indicate that 79 and 73% of the population have limited health literacy in Latvia and Lithuania, respectively. Therefore, the problem of insufficient health literacy is more pronounced in these countries than in other European countries [3].

The most important determinants of the health literacy level, as revealed by the Spearman correlations and multiple regressions analyses, include age, financial situation, social status, and ethnicity. In particular, elderly (aged 76 and over) and the Latvian-speaking population are less health literate, while those having better financial situation and higher social status are more health literate. In addition to these determinants, during the in-depth interviews the healthcare industry experts emphasized the importance of gender and education. In particular, they argued that women tend to be more health literate than men, and those with higher education can better understand, apply, and appraise information relevant to health than individuals with lower levels of education. Furthermore, the experts listed four additional determinants not covered in the previous literature and thus not investigated in the survey: access to health services; ability to access health-related information; traditions affecting the new generations’ health-related behaviour (e.g., eating habits, use of non-prescription drugs); and the value and priority of health as perceived by the individuals*.*

Given the limited level of health literacy in Latvia and Lithuania, it is imperative to develop policies for improving it. First, as suggested by the experts, health education should be improved. Currently, parents are typically unable to provide children with basic knowledge about health. By providing health education in schools, the quality of health-related information passed down from generation to generation would improve in the long run. Second, as there is a lack of structured health-related information in Latvian, increasing its availability might positively influence the level of health literacy. Finally, a database with guidelines explaining how to deal with the most common health-related problems would help to decrease negative consequences of self-medication and, at the same time, increase the health literacy of the general population.

## Supplementary Information

**Additional file 1.**

**Additional file 2.**

**Additional file 3.**

## Data Availability

The survey data from the paper-assisted personal interviews is provided as an Online Supplementary Material S3. The anonymous data file includes the demographic and socioeconomic characteristics of the respondents’, and the answers to the 47-item HLS-EU-Q questionnaire. The transcripts of the interviews with the healthcare industry experts are available from the corresponding author upon reasonable request in Latvian, Lithuanian or Russian, depending on the choice of the interviewee.

## Abbreviations

EU: European Union
HLS-EU: European Health Literacy Survey
HLS-EU-Q: European Health Literacy Questionnaire
PAPI: Paper assisted personal interview
UNESCO: United Nations Educational, Scientific and Cultural Organization
UK: United Kingdom
US: United States
ISCED: International Standard Classification of Education
VIF: Variance inflation factor
WHO: World Health Organization
