# Supplementary material for: Level of health literacy in Latvia and Lithuania: a population-based study
Source: Arch Public Health. 2022 Jul 11;80:166. doi: 10.1186/s13690-022-00886-3 (PMC9275389; doi:10.1186/s13690-022-00886-3)
Supplement: Supplementary file 1 — Additional file 1. [file 13690_2022_886_MOESM1_ESM.pdf]

Electronic Supplementary Material S1: Health literacy survey

Questionnaire – English Version

Health Literacy Survey

Stockholm School of Economics in Riga

We are Year 3 students from the Stockholm School of Economics in Riga researching health literacy topic for our Bachelor Thesis. The aim of this survey is to investigate health literacy level and its determinants in Latvia and Lithuania to contribute to the improvement of healthcare systems in these countries. The survey has been already carried out in several other European countries.

Your participation in this research is voluntary. You may choose not to participate. The procedure involves answering survey questions that will take approximately 15 minutes. Your responses will be treated confidentially, no identifying information such as name, personal number and other will be collected. The results of this study will be used for scholarly purposes only.

If you are at least 18 years old and you agree to the statement above, please mark "Agree":

- ☐ Agree

Part 1: General information

1. What is your gender?
   - ☐ Male
   - ☐ Female
   - ☐ Other____

2. What is your age?
   - ☐ 18 - 25
   - ☐ 26 - 35
   - ☐ 36 - 45
   - ☐ 46 - 55
   - ☐ 56 - 65
   - ☐ 66 - 75
   - ☐ 76 and older

3. What is your level of education?
   - ☐ No school education
   - ☐ Primary education
   - ☐ Lower secondary education (Latvia: 9-year basic education; Lithuania: 10-year basic education)
   - ☐ Upper secondary education (academic secondary education/ 12-year basic education)
   - ☐ Post-secondary education (vocational secondary education; professional qualification)
   - ☐ Higher education (bachelor's degree or master's degree)

- ☐ Post graduate education (doctor’s degree)

4. What is your status of employment?
   - ☐ Full-time
   - ☐ Part-time
   - ☐ Unemployed
   - ☐ Retired
   - ☐ Other____

5. How would you assess your financial situation?
   - ☐ Very poor
   - ☐ Below average
   - ☐ Average
   - ☐ Above average
   - ☐ Excellent

6. How would you assess your social status?
   - ☐ Very low
   - ☐ Low
   - ☐ Lower middle
   - ☐ Middle
   - ☐ Upper middle
   - ☐ High
   - ☐ Very high

7. How frequently did you use health services in the last 12 months (e.g. emergency services, hospital services, doctor visits, etc.)?
   - ☐ 0 times
   - ☐ 1 – 4 times
   - ☐ 5 – 8 times
   - ☐ 9 times and more

Part 2: Health Literacy Survey

	Very difficult	Fairly difficult	Fairly easy	Very easy
On a scale from *Very difficult* to *Very easy*, how easy would you say it is to…				
**Subscale: HEALTH CARE**				
1) ... find information about symptoms of illnesses that concern you?				
2) …find information on treatments of illnesses that concern you?				
3) …find out what to do in case of a medical emergency?				
4) …find out where to get professional help when you are ill? *(Instructions: such as doctor, pharmacist, psychologist)*				
5) …understand what your doctor says to you?				
6) …understand the leaflets that come with your medicine?				
7) …understand what to do in a medical emergency?				
8) …understand your doctor’s or pharmacist’s instructions on how to take a prescribed medicine?				

9) …judge how information from your doctor applies to you?				
10) …judge the advantages and disadvantages of different treatment options?				
11) …judge when you may need to get a second opinion from another doctor?				
12) …judge if the information about illness in the media is reliable? *(Instructions: TV, Internet or other media)*				
13) …use information the doctor gives you to make decisions about your illness?				
14) …follow the instructions on medication?				
15) …call an ambulance in an emergency?				
16) …follow instructions from your doctor or pharmacist?				
**Subscale: DISEASE PREVENTION**				
17) …find information about how to manage unhealthy behaviour such as smoking, low physical activity and drinking too much?				
18) …find information on how to manage mental health problems like stress or depression?				
19) …find information about vaccinations and health screenings that you should have? *(Instructions: breast exam, blood sugar test, blood pressure)*				
20) …find information on how to prevent or manage conditions like being overweight, high blood pressure or high cholesterol?				
21) …understand health warnings about behaviour such as smoking, low physical activity and drinking too much?				
22) …understand why you need vaccinations?				
23) …understand why you need health screenings? *(Instructions: breast exam, blood sugar test, blood pressure)*				
24) …judge how reliable health warnings are, such as smoking, low physical activity and drinking too much?				
25) …judge when you need to go to a doctor for a check-up?				
26) …judge which vaccinations you may need?				
27) …judge which health screenings you should have? *(Instructions: breast exam, blood sugar test, blood pressure)*				
28) …judge if the information on health risks in the media is reliable? (*Instructions: TV, Internet or other media)*				
29) …decide if you should have a flu vaccination?				
30) …decide how you can protect yourself from illness based on advice from family and friends?				
31) …decide how you can protect yourself from illness based on information in the media? *(Instructions: Newspapers, leaflets, Internet or other media)*				
**Subscale: HEALTH PROMOTION**				
32) …find information on healthy activities such as exercise, healthy food and nutrition?				
33) …find out about activities that are good for your mental well-being? *(Instructions: meditation, exercise, walking, pilates* etc.*)*				

34) …find information on how your neighborhood could be more health-friendly? *(Instructions: Reducing noise and pollution, creating green spaces, leisure facilities)*				
35) …find out about political changes that may affect health? *(Instructions: legislation, new health screening programmes, changing of government, restructuring of health services* etc.*)*				
36) …find out about efforts to promote your health at work?				
37) …understand advice on health from family members or friends?				
38) …understand information on food packaging?				
39) …understand information in the media on how to get healthier? *(Instructions: Internet, newspapers, magazines)*				
40) …understand information on how to keep your mind healthy?				
41) …judge where your life affects your health and well-being? *(Instructions: Your community, your neighbourhood)*				
42) …judge how your housing conditions help you to stay healthy?				
43) …judge which everyday behavior is related to your health? *(Instructions: Drinking and eating habits, exercise* etc.*)*				
44) …make decisions to improve your health?				
45) …join a sports club or exercise class if you want to?				
46) …influence your living conditions that affect your health and well-being? *(Instructions: Drinking and eating habits, exercise* etc.*)*				
47) …take part in activities that improve health and well-being in your community?				

Questionnaire – Latvian Version

Veselības Izglītības Aptauja
Rīgas Ekonomikas augstskola

Mēs esam 3. kursa studenti no Rīgas Ekonomikas augstskolas, kas pēta veselības izglītības tēmu bakalaura darba ietvaros. Šīs aptaujas mērķis ir noskaidrot veselības izglītības līmeni un to noteicošos faktorus Latvijā un Lietuvā, lai sniegtu ieguldījumu veselības aprūpes sistēmu uzlabošanā šajās valstīs. Aptauja jau ir veikta vairākās citās Eiropas valstīs.

Jūsu dalība šajā pētījumā ir brīvprātīga. Jūs varat izvēlēties nepiedalīties. Procedūra ietver aptauju, kas prasīs aptuveni 15 minūtes. Jūsu atbildes būs konfidenciālas, un mēs neapkoposim identificējošu informāciju, piemēram, vārdu, personas kodu u.t.t. Šī pētījuma rezultāti tiks izmantoti tikai zinātniskiem mērķiem.

Ja esat vismaz 18 gadus vecs un piekrītat iepriekš norādītajai informācijai, lūdzu, atzīmējiet "Piekrītu":

- ☐ Piekrītu

1.daļa: Vispārīga informācija

1. Kāds ir Jūsu dzimums?
   - ☐ Vīrietis
   - ☐ Sieviete
   - ☐ Cits____

2. Kāds ir Jūsu vecums?
   - ☐ 18 - 25
   - ☐ 26 - 35
   - ☐ 36 - 45
   - ☐ 46 - 55
   - ☐ 56 - 65
   - ☐ 66 - 75
   - ☐ 76 un vairāk

3. Kāds ir Jūsu izglītības līmenis?
   - ☐ Pirmsskolas izglītība
   - ☐ Sākumsskolas izglītība
   - ☐ Pamatizglītība
   - ☐ Vidējā izglītība
   - ☐ Profesionālā izglītība
   - ☐ Augstākā izglītība (bakalaura vai maģistra grāds)
   - ☐ Augstākā izglītība (doktora grāds)

4. Kāds ir Jūsu nodarbinātības statuss?
   - ☐ Pilna laika
   - ☐ Nepilna laika
   - ☐ Bezdarbnieks
   - ☐ Atvuļināts

- ☐ Cits____

5. Kā Jūs novērtētu savu finansiālo stāvokli?
   - ☐ Ļoti slikts
   - ☐ Zem vidējā līmeņa
   - ☐ Vidējāis
   - ☐ Virs vidējā līmeņa
   - ☐ Augsts

6. Kā Jūs novērtētu savu sociālo stāvokli?
   - ☐ Ļoti zems
   - ☐ Zems
   - ☐ Zem vidējā līmeņa
   - ☐ Vidējāis
   - ☐ Virs vidējā līmeņa
   - ☐ Augsts
   - ☐ Ļoti augsts

7. Cik bieži pēdējos 12 mēnešu laikā Jūs izmantojāt veselības pakalpojumus (piemēram, neatliekamās palīdzības dienesta pakalpojumus, slimnīcas pakalpojumus, ārstu pakalpojumus u.t.t.)?
   - ☐ 0 reizes
   - ☐ 1 – 4 reizes
   - ☐ 5 – 8 reizes
   - ☐ 9 reizes un vairāk

2.daļa: Veselības izglītības aptauja

	Ļoti grūti	Diezgan grūti	Viegli	Ļoti viegli
Skalā no *Ļoti grūti* līdz *Ļoti viegli*, novērtē, cik viegli ir …				
**Apakšskala: VESELĪBAS APRŪPE**				
48) …atrast informāciju par slimības simptomiem, kas skar Jūs?				
49) … atrast informāciju par slimības ārstēšanu, kas skar Jūs?				
50) …atrast informāciju, ko darīt ārkārtas situācijas gadījumā?				
51) …atrast speciālistu, kuru apmeklēt slimības gadījumā? *(piemēram: ārsts, farmaceits, psihologs)*				
52) …saprast ko ārsta ieteikumus?				
53) …saprast instrukcijas, kas nāk klāt medikamentiem?				
54) …saprast, ko darīt ārkārtas situācijas gadījumā?				

55) …saprast ārsta vai farmaceita norādes par izrakstītajām zālēm?				
56) …izvērtēt, kā Jūs ietekmē ārsta sniegtā informācija?				
57) …izvērtēt priekšrocības un trūkumus dažādām ārstniecības iespējām?				
58) …izvērtēt, kad ir nepieciešams saņemt papildus ieteikumu no cita ārsta?				
59) …saprast, vai informācija par slimībām medijos ir uzticama? *(piemēram: TV, internets u.c. mediji)*				
60) …izmantot ārsta sniegto informāciju, lai pieņemtu lēmumus par slimību?				
61) …sekot līdzi medikamentu instrukcijai?				
62) …zvanīt ātrajai palīdzībai ārkārtas situācijā?				
63) … sekot līdzi ārsta vai farmaceita instrukcijai?				
**Apakšskala: SLIMĪBU PROFILAKSE**				
64) …atrast informāciju kā pārvaldīt neveselīgus ieradumus kā smēķēšana, mazkustīgs dzīvesveids un alkoholisms?				
65) …atrast informāciju kā pārvaldīt mentālās veselības kaites kā stress vai depresija?				
66) …atrast informāciju par vakcīnām un regulārajām veselības pārbaudēm? (*piemēram: asinsanalīzes, cukura līmenis asinīs u.t.t.*)				
67) …atrast informāciju kā novērst vai pārvaldīt veselības problēmas kā aptaukošanās, paaugstināts asinsspiediens vai augsts holesterīns?				
68) …saprast brīdinājumus par ietekmi uz veselību no aktivitātēm kā smēķēšana, mazkustīgs dzīvesveids un alkoholisms?				
69) …saprast, kādēļ ir nepieciešama vakcinācija?				
70) … saprast, kādēļ ir nepieciešamas veselības pārbaudes (*piemēram: asinsanalīzes, cukura līmenis asinīs u.t.t.*)				
71) …izvērtēt, cik ticami ir brīdinājumi par ietekmi uz veselību no aktivitātēm kā smēķēšana, mazkustīgs dzīvesveids, alkoholisms?				
72) …izvērtēt, kad ir nepieciešama ārsta vizīte?				
73) …izvērtēt, kādas vakcīnas būtu nepieciešamas?				
74) …izvērtēt, kādas veselības pārbaudes būtu nepieciešamas? (*piemēram: asinsanalīzes, cukura līmenis asinīs u.t.t.*)				
75) …izvērtēt cik ticama ir informācija par veselības riskiem medijos? *(piemēram: TV, internets u.c. mediji)*				

76) …izlemt, vai gripas vakcīna ir nepieciešama?				
77) …izlemt, kā sevi pasargāt no saslimšanas balstoties uz ieteikumiem no draugiem un ģimenes?				
78) … izlemt, kā sevi pasargāt no saslimšanas balstoties uz informāciju medijos? (*piemēram: laikraksti, brošūras, internets u.c. mediji)*				
**Apakšskala: VESELĪBAS VEICINĀŠANA**				
79) …atrast informāciju par veselīgu dzīvesveidu, piemēram, veselīga pārtika un uzturs?				
80) …atrast aktivitātes, kas veicina garīgo labklājību?				
81) …atrast informāciju kā uzlabot apkārtējo vidi, padarīt to dabai draudzīgāku? (*piemēram: piesārņojuma samazināšana, zaļo zonu ieviešana, atpūtas zonas)*				
82) …atrast informāciju par politiskām izmaiņām, kas varētu ietekmēt veselību? (*piemēram: izmaiņas likumdošanā, jaunas veselības pārbaudes programmas, izmaiņas valdībā, veselības pakalpojumu sniedzēju restrukturizācija u.t.t.)*				
83) …atrast informāciju par veidiem, kā uzlabot veselību darba vidē?				
84) …saprast ieteikumus par veselību no ģimenes locekļiem vai draugiem?				
85) …saprast informāciju uz pārtikas iepakojumiem?				
86) …saprast informāciju medijos kā uzlabot veselības stāvokli? (*piemēram: internetā, avīzēs, žurnālos u.c. medijos)*				
87) …saprast informāciju kā uzturēt garīgo veselību?				
88) …izvērtēt kā vide ietekmē Jūsu veselību un labklājību? (*piemēram: kopiena, ar ko pavadi laiku, vide, kurā dzīvo)*				
89) …izvērtēt, kā mājas apstākļi palīdz palikt veselam?				
90) …izvērtēt, kuri ikdienas paradumi ietekmē Jūsu veselību? (*piemēram: ēšanas un dzeršanas ieradumi, sporta nodarbības u.c.)*				
91) …pieņemt lēmumu, lai uzlabotu veselību?				
92) …pievienoties sporta klubam vai citām sporta nodarbībām?				
93) …ietekmēt dzīves apstākļus, kas iespaido veselību un labklājību? *(piemēram: ēšanas un dzeršanas ieradumi, sporta nodarbības u.c.)*				
94) …piedalīties aktivitātēs, kas uzlabo veselību un labklājību kopienā?				

Questionnaire – Lithuanian Version

Europos sveikatos raštingumo klausimynas
Stockholm School of Economics in Riga

Esame Stockholm School of Economics in Riga universiteto 3 kurso studentės, tiriančios sveikatos raštingumo lygį savo bakalauro darbui. Šios apklausos tikslas – išsiaiškinti sveikatos raštingumo lygį ir jį lemiančius veiksnius Lietuvoje ir prisidėti prie sveikatos priežiūros sistemos tobulinimo šalyje. Toks pat tyrimas buvo atliktas 8 Europos Sąjungos šalyse.

Jūsų dalyvavimas šiame tyrime yra savanoriškas. Galite pasirinkti nedalyvauti. Ši apklausa užtruks apie 15 minučių. Jūsų atsakymai bus konfidencialūs ir mes nerinksime tokios asmeninės informacijos, kaip vardas, asmens kodas ir pan. Šio tyrimo rezultatai bus naudojami tik mokslo tikslams.

Jeigu esate vyresnis/vyresnė nei 18 metų ir sutinkate su aukščiau pateikta informacija, prašome pažymėti „Sutinku“:

- ☐ Sutinku

Pirmoji dalis: Bendroji informacija

1. Kokia yra jūsų lytis?
   - ☐ Vyras
   - ☐ Moteris
   - ☐ Kita:____

2. Koks yra jūsų amžius?
   - ☐ 18 - 25
   - ☐ 26 - 35
   - ☐ 36 - 45
   - ☐ 46 - 55
   - ☐ 56 - 65
   - ☐ 66 - 75
   - ☐ 76 ir vyresnis/vyresnė

3. Koks yra jūsų išsilavinimas?
   - ☐ Be išsilavinimo
   - ☐ Pradinis išsilavinimas
   - ☐ Pagrindinis išsilavinimas (10-metų pagrindinis išsilavinimas)
   - ☐ Vidurinis išsilavinimas
   - ☐ Aukštasis neuniversitetinis išsilavinimas/ Profesinis išsilavinimas
   - ☐ Aukštasis universitetinis išsilavinimas (bakalauro arba magistro laipsnis)
   - ☐ Mokslinis laipsnis

4. Koks yra jūsų užimtumo statusas?
   - ☐ Darbas pilnu etatu
   - ☐ Darbas nepilnu etatu
   - ☐ Bedarbis/bedarbė

- Pensininkas/pensininkė
- Kita: ____

5. Kaip įvertintumėte savo finansinę padėtį?
   - Labai prasta
   - Žemiau vidutinės
   - Vidutinė
   - Aukščiau vidutinės
   - Puiki

6. Kaip įvertintumėte savo socialinį statusą?
   - Labai žemas
   - Žemas
   - Truputį žemesnis nei vidutinis
   - Vidutinis
   - Truputį aukštesnis nei vidutinis
   - Aukštas
   - Labai aukštas

7. Kaip dažnai per pastaruosius 12 mėnesių naudojotės sveikatos priežiūros paslaugomis (pvz. skubios pagalbos paslaugomis, ligoninių teikiamomis paslaugomis, kreipėtės į gydytoją ir pan.)?
   - Nesinaudojau
   - 1 – 4 kartus
   - 5 – 8 kartus
   - 9 kartus ir daugiau

Antroji dalis: Europos sveikatos raštingumo klausimynas (HLS-EU-Q47)

	Labai sunku	Pakankamai sunku	Pakankamai lengva	Labai lengva
Kaip manote (skalėje nuo labai sunku iki labai lengva), kaip lengva yra:				
**Potemė: SVEIKATOS APSAUGA**				
1) ... rasti informacijos apie ligos, kuri jus neramina, simptomus?				
2) … rasti informacijos apie ligos, kuri jus neramina, gydymą?				
3) … sužinoti, ką daryti nelaimės atveju?				
4) … sužinoti, kur kreiptis profesionalios pagalbos, kai sergate? *(Instrukcijos: gydytojas, vaistininkas, psichologas)*				
5) … suprasti, ką sako gydytojas?				
6) … suprasti pakuotės lapelius, kurie pateikiami kartu su jūsų vaistais?				
7) … suprasti, ką daryti nelaimės atvejais?				
8) … suprasti gydytojo ar vaistininko pateiktas instrukcijas/nurodymus, kaip vartoti išrašytus vaistus?				
9) … nuspręsti, kaip jūsų gydytojo pateikta informacija taikoma jums?				

10) … įvertinti, kokie yra skirtingų gydymo metodų privalumai ir trūkumai?				
11) … nuspręsti, kada gali prireikti antros kito gydytojo nuomonės?				
12) … įvertinti, ar viešojoje erdvėje (pvz. televizijoje, internete ir pan.) pateikiama informacija apie ligas yra patikima?				
13) … naudotis informacija, kurią gydytojas pateikia, jums norint priimti sprendimus, susijusius su jūsų liga?				
14) … laikytis gydymo ir vaistų vartojimo instrukcijų?				
15) … išsikviesti greitąją pagalbą nelaimės atveju?				
16) … laikytis gydytojo arba vaistininko nurodymų?				
**Potemė: LIGŲ PREVENCIJA**				
17) … rasti informacijos apie tai, kaip riboti žalingus įpročius, pvz. rūkymą, mažą fizinį aktyvumą ir alkoholio vartojimą?				
18) … rasti informacijos apie tai, kaip spręsti psichologines problemas, pvz. mažinti stresą ir depresiją?				
19) … rasti informacijos apie skiepus ir sveikatos patikrinimus, kuriuos turėtumėte atlikti? *(Instrukcijos: krūtų tyrimas, cukraus kiekio kraujyje tyrimas, kraujospūdis)*				
20) … rasti informacijos apie tai, kaip užkirsti kelią antsvoriui, aukštam kraujospūdžiui ar padidėjusiam cholesterolio kiekiui?				
21) … suprasti įspėjimus apie pakitusią sveikatos būklę dėl tokio elgesio kaip rūkymas, mažas fizinis aktyvumas ir per didelis alkoholio vartojimas?				
22) … suprasti, kodėl jums reikalingi skiepai?				
23) …suprasti, kodėl jums reikalingas sveikatos patikrinimas? *(Instrukcijos: krūtų tyrimas, cukraus kiekio kraujyje tyrimas, kraujo spaudimas)*				
24) … nuspręsti, kiek patikimi yra įspėjimai apie sveikatą, pvz. rūkymas, mažas fizinis aktyvumas ir per didelis alkoholio vartojimas?				
25) … nuspręsti, kada jums reikia kreiptis į gydytoją norint atlikti sveikatos apžiūrą?				
26) … nuspręsti, kokių skiepų jums gali prireikti?				
27) …nuspręsti, kokius sveikatos patikrinimus turėtumėte atlikti? *(Instrukcijos: krūtų tyrimas, cukraus kiekio kraujyje tyrimas, kraujospūdis)*				
28) … nuspręsti, ar žiniasklaidos pateikiama informacija apie pavojų sveikatai yra patikima? *(Instrukcijos: televizija, internetas ar pan.)*				
29) … nuspręsti, ar reikia skiepytis nuo gripo?				
30) … remiantis šeimos ir draugų patarimais nuspręsti, kaip galite apsisaugoti nuo ligos?				
31) … nuspręsti, kaip galite apsisaugoti nuo ligų, remdamiesi žiniasklaidos informacija? *(Instrukcijos: laikraščiai, lankstinukai, internetas ar pan.)*				

**Potemė: SVEIKATOS STIPRINIMAS**				
32) … rasti informacijos apie sveiką gyvenseną, pvz. mankštą, sveiką maistą ir mitybą?				
33) … sužinoti apie veiklą, naudingą jūsų psichologinei gerovei? *(Instrukcijos: meditacija, mankšta, vaikščiojimas, pilatesas ir pan.)*				
34) … rasti informacijos apie tai, kaip jūsų kaimynystė galėtų būti palankesnė sveikatai? *(Instrukcijos: triukšmo ir taršos mažinimas, žaliųjų erdvių bei laisvalaikio įrenginių kūrimas)*				
35) … sužinoti apie politinius pokyčius, kurie gali turėti įtakos sveikatai? *(Instrukcijos: įstatymai, naujos sveikatos patikrinimo programos, vyriausybės keitimas, sveikatos paslaugų pertvarkymas ir pan.)*				
36) … sužinoti apie pastangas gerinti savo sveikatą darbe?				
37) … suprasti šeimos narių ar draugų patarimus sveikatos klausimais?				
38) … suprasti informaciją, esančią ant maisto pakuočių?				
39) … suprasti žiniasklaidos informaciją apie tai, kaip sveikiau gyventi? *(Instrukcijos: internetas, laikraščiai, žurnalai)*				
40) … suprasti informaciją apie tai, kaip išlaikyti sveiką psichologinę būseną?				
41) … nuspręsti, kada jūsų gyvenimas daro įtaką jūsų sveikatai ir gerovei*? (Instrukcijos: jūsų bendruomenė, jūsų kaimynystė)*				
42) … nuspręsti, kaip jūsų būsto sąlygos padeda jums išlikti sveikiems?				
43) … nuspręsti, koks kasdienis elgesys yra susijęs su jūsų sveikata? *(Instrukcijos: gėrimo ir mitybos įpročiai, mankšta ir pan.)*				
44) … priimti sprendimus norint pagerinti savo sveikatą?				
45) … pradėti lankyti sporto klubą ar mankštos užsiėmimus?				
46) … paveikti jūsų gyvenimo sąlygas, kurios turi įtakos jūsų sveikatai ir gerovei? *(Instrukcijos: gėrimo ir mitybos įpročiai, mankšta ir pan.)*				
47) … dalyvauti veikloje, kuri gerina jūsų bendruomenės sveikatą ir gerovę?				

Questionnaire – Russian Version

Анкета по исследованию грамотности населения в области здравоохранения
Стокгольмская школа экономики в Риге

Мы студенты третьего курса Стокгольмской школы экономики в Риге (SSE Riga), изучающие тему грамотности населения в области здравоохранения в рамках нашей дипломной работы. Целью данного опроса является определение уровня грамотности в области здравоохранения и его определяющих факторов в Латвии и Литве, чтобы способствовать улучшению систем здравоохранения в этих странах. Данный опрос уже проводился в других Европейских странах.

Ваше участие в этом исследовании является добровольным. Вы можете отказаться от участия. Процедура включает в себя опрос, который займет около 15 минут. Ваши ответы будут конфиденциальными, и идентифицирующая информация, такая как имя, персональный код и другие не будут собраны. Результаты этого исследования будут использоваться только в научных целях.

Если Вам не менее 18 лет, и Вы согласны с указанной выше информацией, просим отметить «Согласен(а)»:

- ☐ Согласен(а)

Часть 1: Общая информация

8. Укажите Ваш пол
   - ☐ Мужской
   - ☐ Женский
   - ☐ Другой ____

9. Ваш возраст
   - ☐ 18 - 25
   - ☐ 26 - 35
   - ☐ 36 - 45
   - ☐ 46 - 55
   - ☐ 56 - 65
   - ☐ 66 - 75
   - ☐ 76 и старше

10. Ваше образование
    - ☐ Дошкольное образование
    - ☐ Начальное образование
    - ☐ Основное образование
    - ☐ Среднее образование

- Профессиональное образование
- Высшее образование (степень бакалавра или магистра)
- Высшее образование (докторская степень)

11. Каков Ваш статус занятости?
    - Работа на полную ставку
    - Работа на неполную ставку
    - Безработный
    - На пенсии
    - Другой____

12. Как бы Вы оценили свое финансовое положение?
    - Недостаточное
    - Ниже среднего
    - Среднее
    - Выше среднего
    - Отличное

13. Как бы Вы оценили свой социальный статус?
    - Очень низкий
    - Низкий
    - Ниже среднего
    - Средний
    - Выше среднего
    - Высокий
    - Очень высокий

14. Как часто Вы пользовались медицинскими услугами за последние 12 месяцев (например, вызывали неотложную медицинскую помощь, пользовались услугами больницы, посещали врача и другие)?
    - 0 раз
    - 1 – 4 раза
    - 5 – 8 раз
    - 9 раз и более

Часть 2: Анкета по исследованию грамотности населения в области здравоохранения

	Крайне сложно	Довольно сложно	Легко	Очень легко
Как бы Вы оценили вопрос по шкале от *Крайне сложно* до *Очень легко*:				

**Область: ЗДРАВООХРАНЕНИЕ**				
95) …найти информацию о симптомах заболеваний, которые Вас беспокоят?				
96) …найти информацию о лечении заболеваний, которые Вас беспокоят?				
97) …найти информацию о том, как действовать в случае необходимости воспользоваться неотложной медицинской помощью?				
98) …найти информацию о том, где можно получить профессиональную медицинскую помощь в случае заболевания? *(например: врач, фармацевт, психолог)*				
99) …понимать, что рекомендует лечащий врач?				
100) …понимать инструкцию по применению лекарств?				
101) ... понимать, как действовать в случае неотложной медицинской помощи?				
102) …понимать рекомендации лечащего врача или фармацевта о том как необходимо принимать назначенные лекарства?				
103) … оценить насколько полученная информация/рекомендация от лечащего врача применима к Вам?				
104) …оценить преимущества и недостатки различных вариантов лечения?				
105) …оценить в каком случае необходимо получить альтернативное мнение от другого лечащего врача?				
106) …оценить насколько надежна информацию о заболеваниях в СМИ? *(например: телевидение, Интернет или другие СМИ)*				
107) …применять информацию, полученную от лечащего врача, чтобы сделать выводы о степени заболевания?				
108) …следовать инструкции по применению лекарств?				
109) …вызвать неотложную скорую помощь?				
110) …следовать рекомендациям лечащего врача или фармацевта?				
**Область: ПРОФИЛАКТИКА ЗАБОЛЕВАНИЙ**				
111) …найти информацию о том, как справиться с такими проблемами как курение, низкая физическая активность и чрезмерное употребление алкоголя?				

112) …найти информацию о том, как справиться с такими проблемами психологического характера как стресс или депрессия?				
113) …найти информацию о необходимых прививках и медицинских обследованиях? *(например: обследование грудной клетки, анализ уровня сахара в крови, артериальное давление)*				
114) … найти информацию о том, как предотвратить или справиться с такими проблемами как избыточный вес, высокое артериальное давление или высокий уровень холестерина?				
115) …понимать к каким последствиям может привести курение, недостаточная физическая активность и чрезмерное употребление алкоголя?				
116) …понимать для чего необходимы прививки?				
117) …понимать для чего необходимы медицинские обследования? *(например: обследование грудной клетки, анализ уровня сахара в крови, артериальное давление)*				
118) … оценить насколько достоверны предупреждения о вреде для здоровья от курения, недостаточной физической активности и чрезмерного употребления алкоголя?				
119) …оценить, когда необходимо посетить лечащего врача для обследования?				
120) …оценить какие прививки Вам необходимы?				
121) …оценить какие медицинские обследования Вам необходимы? (*например: обследование грудной клетки, анализ уровня сахара в крови, артериальное давление)*				
122) … оценить насколько надежна информация о рисках для здоровья в СМИ? *(например: телевидение, Интернет или другие СМИ)*				
123) …решить, есть ли необходимость делать прививку от гриппа?				
124) …решить, как можно предотвратить возможность заболеваний, основываясь на советах от семьи и друзей?				
125) … решить, как можно предотвратить возможность заболеваний, основываясь на информации из СМИ? *(например: газеты, брошюры, Интернет и другие СМИ)*				
**Область: УКРЕПЛЕНИЕ ЗДОРОВЬЯ**				
126) …найти информацию о физических упражнениях и здоровом питании?				
127) …найти информацию об активностях, которые положительно влияют на ментальное				

благосостояние? *(например: медитация, упражнения, пешие прогулки, пилатес и т.д.)*				
128) …найти информацию о том, как место проживания/район может стать более благоприятным для здоровья? *(например: снижение уровня шума и загрязнений, создание зеленых зон и объектов для отдыха)*				
129) …найти информация о политических решениях, которые могут повлиять на сферу здравоохранения? *(например: законодательство, новые программы медицинских услуг, изменение в правительстве, реструктуризация медицинских услуг и т.д.)*				
130) …найти информацию о способах укрепления здоровья на рабочем месте?				
131) …понимать советы друзей и близких по теме здоровья?				
132) …понимать информацию на упаковках продуктов питания?				
133) …понимать информацию полученную из СМИ об укреплении здоровья? *(например: Интернет, газеты, журналы)*				
134) …понимать информацию о том, как сохранить/укрепить ментальное здоровье?				
135) …оценить как Ваш образ жизни влияет на здоровье и благополучие? *(например: окружение)*				
136) …оценить как Ваши условия проживания сказываются на состоянии здоровья?				
137) …оценить как повседневное поведение влияет на состояние здоровья? *(например: привычки, упражнения и т.д.)*				
138) …принимать решения положительно сказывающиеся на состоянии здоровья?				
139) …начать посещать спортивный зал или групповые тренировки/занятия при необходимости?				
140) …повлиять на условия жизни, которые скажутся на состоянии здоровья и благополучии? *(например: привычки, упражнения и т.д.)*				
141) …принимать участие в мероприятиях, которые улучшают состояние здоровья и благополучие Вашего окружения?				
