# Supplementary material for: Level of health literacy in Latvia and Lithuania: a population-based study
Source: Arch Public Health. 2022 Jul 11;80:166. doi: 10.1186/s13690-022-00886-3 (PMC9275389; doi:10.1186/s13690-022-00886-3)
Supplement: Supplementary file 2 — Additional file 2. [file 13690_2022_886_MOESM2_ESM.pdf]

Electronic Supplementary Material S2. Distribution of responses in the 47-item European Health Literacy Questionnaire

Latvia

Questions	Very difficult	Fairly difficult	Fairly easy	Very easy	Do not know
Q1	1%	26%	67%	6%	0%
Q2	3%	37%	55%	5%	0%
Q3	2%	25%	54%	19%	0%
Q4	5%	35%	49%	12%	0%
Q5	3%	16%	70%	11%	0%
Q6	4%	14%	65%	17%	0%
Q7	6%	36%	53%	5%	0%
Q8	0%	9%	76%	15%	0%
Q9	5%	34%	54%	7%	0%
Q10	11%	50%	36%	3%	0%
Q11	9%	53%	34%	4%	0%
Q12	18%	54%	24%	4%	0%
Q13	3%	34%	57%	5%	1%
Q14	2%	12%	62%	24%	0%
Q15	2%	9%	55%	34%	0%
Q16	0%	9%	69%	21%	1%
Q17	5%	12%	57%	24%	2%
Q18	8%	29%	51%	11%	1%
Q19	7%	38%	47%	8%	1%
Q20	3%	28%	56%	12%	1%
Q21	0%	10%	64%	24%	2%
Q22	5%	18%	61%	15%	1%
Q23	1%	12%	65%	21%	1%
Q24	1%	24%	55%	18%	2%
Q25	4%	33%	56%	7%	0%
Q26	12%	36%	45%	7%	1%
Q27	10%	41%	43%	6%	1%
Q28	13%	50%	34%	3%	1%
Q29	9%	45%	41%	5%	1%
Q30	5%	40%	48%	7%	1%
Q31	10%	45%	40%	6%	0%
Q32	0%	12%	56%	32%	0%
Q33	5%	15%	52%	28%	0%
Q34	10%	33%	43%	15%	0%
Q35	21%	48%	26%	6%	0%
Q36	4%	42%	43%	12%	0%
Q37	1%	23%	62%	13%	1%
Q38	13%	27%	47%	13%	1%
Q39	2%	31%	55%	11%	1%
Q40	7%	27%	56%	10%	0%
Q41	2%	25%	60%	12%	1%
Q42	3%	33%	52%	12%	0%
Q43	2%	18%	64%	16%	0%
Q44	4%	44%	43%	10%	0%
Q45	11%	43%	34%	13%	0%
Q46	7%	46%	39%	9%	0%
Q47	10%	39%	43%	9%	0%

Lithuania

Questions	Very difficult	Fairly difficult	Fairly easy	Very easy	Do not know
Q1	3%	14%	61%	22%	0%
Q2	3%	22%	65%	8%	2%
Q3	5%	18%	54%	23%	0%
Q4	1%	18%	56%	24%	1%
Q5	1%	10%	73%	15%	1%
Q6	2%	20%	56%	21%	1%
Q7	4%	31%	51%	14%	0%
Q8	1%	5%	63%	31%	0%
Q9	2%	21%	61%	15%	1%
Q10	7%	54%	34%	4%	0%
Q11	6%	48%	41%	5%	0%
Q12	14%	57%	26%	3%	0%
Q13	3%	21%	70%	6%	0%
Q14	1%	9%	60%	29%	0%
Q15	4%	10%	49%	35%	1%
Q16	1%	8%	51%	40%	0%
Q17	0%	9%	48%	42%	1%
Q18	5%	28%	44%	23%	0%
Q19	4%	33%	43%	20%	0%
Q20	3%	24%	50%	23%	0%
Q21	2%	21%	51%	24%	2%
Q22	9%	20%	51%	20%	0%
Q23	2%	7%	55%	35%	0%
Q24	1%	14%	55%	29%	1%
Q25	2%	25%	51%	22%	0%
Q26	16%	45%	32%	6%	1%
Q27	1%	36%	50%	13%	0%
Q28	13%	51%	30%	6%	0%
Q29	16%	35%	32%	16%	1%
Q30	8%	33%	51%	7%	0%
Q31	11%	42%	40%	7%	0%
Q32	3%	9%	48%	40%	0%
Q33	3%	16%	53%	28%	0%
Q34	11%	43%	36%	9%	0%
Q35	16%	54%	26%	4%	0%
Q36	13%	35%	43%	7%	2%
Q37	1%	24%	57%	18%	0%
Q38	19%	32%	40%	9%	0%
Q39	3%	26%	56%	15%	0%
Q40	9%	32%	44%	15%	0%
Q41	3%	24%	61%	11%	0%
Q42	3%	29%	51%	17%	0%
Q43	2%	18%	59%	21%	0%
Q44	1%	34%	49%	16%	0%
Q45	11%	40%	29%	20%	0%
Q46	4%	34%	51%	10%	0%
Q47	7%	38%	43%	13%	0%
